# Equilibrium and Kinetic Studies on Adsorption of Neutral and Ionic Species of Organic Adsorbates from Aqueous Solutions on Activated Carbon

**DOI:** 10.3390/molecules29133032

**Published:** 2024-06-26

**Authors:** Małgorzata Wasilewska, Anna Derylo-Marczewska, Adam W. Marczewski

**Affiliations:** Department of Physical Chemistry, Institute of Chemical Sciences, Maria Curie-Sklodowska University, Maria Curie-Sklodowska Sq. 3, 20-031 Lublin, Poland; awmarcz@wp.pl

**Keywords:** adsorption of aromatic organic pollutants, benzoic acid, nitrobenzoic acids, methylene blue, activated carbon, adsorption equilibrium and kinetics, pH effect

## Abstract

This work presents comprehensive studies of the adsorption of neutral and ionic forms of organic adsorbates from aqueous solutions on activated carbon. The influence of pH on the equilibrium and kinetics of the adsorption of methylene blue (MB) and organic acids, benzoic (BA), 2-nitrobenzoic (2-NBA), 3-nitrobenzoic (3-NBA), and 4-nitrobenzoic (4-NBA) acid, was investigated. Experimental adsorption isotherms were analyzed using the generalized Langmuir isotherm equation (R^2^ = 0.932–0.995). Adsorption rate data were studied using multiple adsorption kinetics equations, of which the multi-exponential equation gave the best fit quality (R^2^ − 1 = (6.3 × 10^−6^)–(2.1 × 10^−3^)). The half-time was also used to represent the effect of pH on adsorption kinetics. Strong dependences of the adsorption efficiency on the solution pH were demonstrated. In the case of organic acid adsorption, the amount and rate of this process increased with a decrease in pH. Moreover, larger adsorbed amounts of methylene blue were recorded in an alkaline environment in a relatively short time. The maximum absorbed amounts were 11.59 mmol/g, 6.57 mmol/g, 9.38 mmol/g, 2.70 mmol/g, and 0.24 mmol/g for BA, 2NBA, 3-NBA, 4-NBA, and MB. The pure activated carbon and the selected samples after adsorption were investigated using thermal analysis and X-ray photoelectron spectroscopy.

## 1. Introduction

Among the substances commonly used in many industrial technologies, the aromatic organic compounds constitute an interesting group. Due to their specific structure, namely the presence of an aromatic ring, they exhibit a number of important properties that can be used in various ways [1,2]. This group of substances includes, among others, benzoic acid and its nitro derivatives (2-, 3-, and 4-nitrobenzoic acids). Benzoic acid is mainly used in the food industry as a preservative or flavor for many food products. This compound and its nitro derivatives are also used for the organic synthesis of many substances, for example alkaloids and pharmaceuticals. An important group of aromatic organic compounds are dyes, including the widely known methylene blue. The latter is used, among others, in industry as a dye, pH indicator, and medicine for fish diseases. Benzoic acid, 2-, 3-, and 4-nitrobenzoic acids, as well as methylene blue are characterized by relatively good solubility in water, which allows free migration. Moreover, the high reactivity of the above-mentioned compounds caused by the presence of an aromatic ring may lead to the formation of new more- or less-toxic compounds [3,4,5,6,7]. Therefore, it is important not to omit this type of substance when purifying water and sewage. As they are highly effective and at the same time neutral for the environment, adsorption methods, using activated carbon as an adsorbent, are an important element of the technology for removing pollutants from water and sewage [8,9,10]. Adsorption is a multi-phase process consisting of stages going on at different time ranges, and the rate of the process is determined by the slowest step. The effectiveness of the adsorption technique is determined by a number of factors, including the following: the properties of the adsorbent [11,12,13] and adsorbate [14,15,16], temperature [17,18,19], mixing speed [20,21,22], the presence of an accompanying substance [23,24,25], and the pH of the solution [26,27,28]. Even a seemingly small increase in the efficiency of adsorption processes used in water and sewage treatment, due to the scale and widespread use of these technologies, may mean achieving a competitive advantage over other solutions. Apart from the cognitive aspect, it is the possible environmental and economic effect that results in ongoing interest in research that will enable deeper knowledge and understanding of the adsorption process, especially adsorption equilibrium and kinetics.

The aim of the work was to study the equilibrium and kinetics of adsorption of neutral and ionic forms of organic pollutants of various acid–base natures. The influence of solution pH on the sorption of benzoic (BA), 2-nitrobenzoic (2-NBA), 3-nitrobenzoic (3-NBA), 4-nitrobenzoic (4-NBA) acids, and methylene blue (MB) was examined. The equilibrium data were described using the Marczewski–Jaroniec isotherm (popularly called the generalized Langmuir (GL) isotherm). The adsorption rate data were analyzed using Bangham plots and the kinetic equations, including the following: the first-order (FOE), the second-order (SOE), the 1,2-mixed-order (MOE), the fractal-like FOE (f-FOE), the fractal-like SOE (f-SOE), the fractal-like MOE (f-MOE), and the multi-exponential (m-exp). Moreover, the interactions in adsorption systems with aromatic organic acids were determined using thermal analysis in an inert gas atmosphere. Finally, the adsorption mechanism for nitrobenzoic acids on GAC 1240W (GAC) activated carbon was assessed using X-ray photoelectron spectroscopy. It should be noted that the research methodology used made it possible to obtain high-quality adsorption data. Particular attention should be paid to kinetic curves that contain up to 100 experimental points. Moreover, in the literature on the subject there are no comprehensive studies describing the adsorption of nitrobenzoic acids substituted in all possible combinations of the position of the substituents—ortho, meta, and para.

## 2. Results

### 2.1. Adsorption Equilibrium

**Figure 1:**
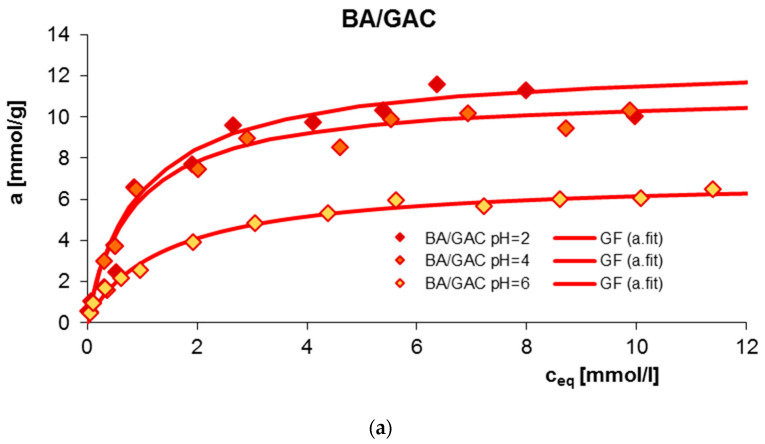

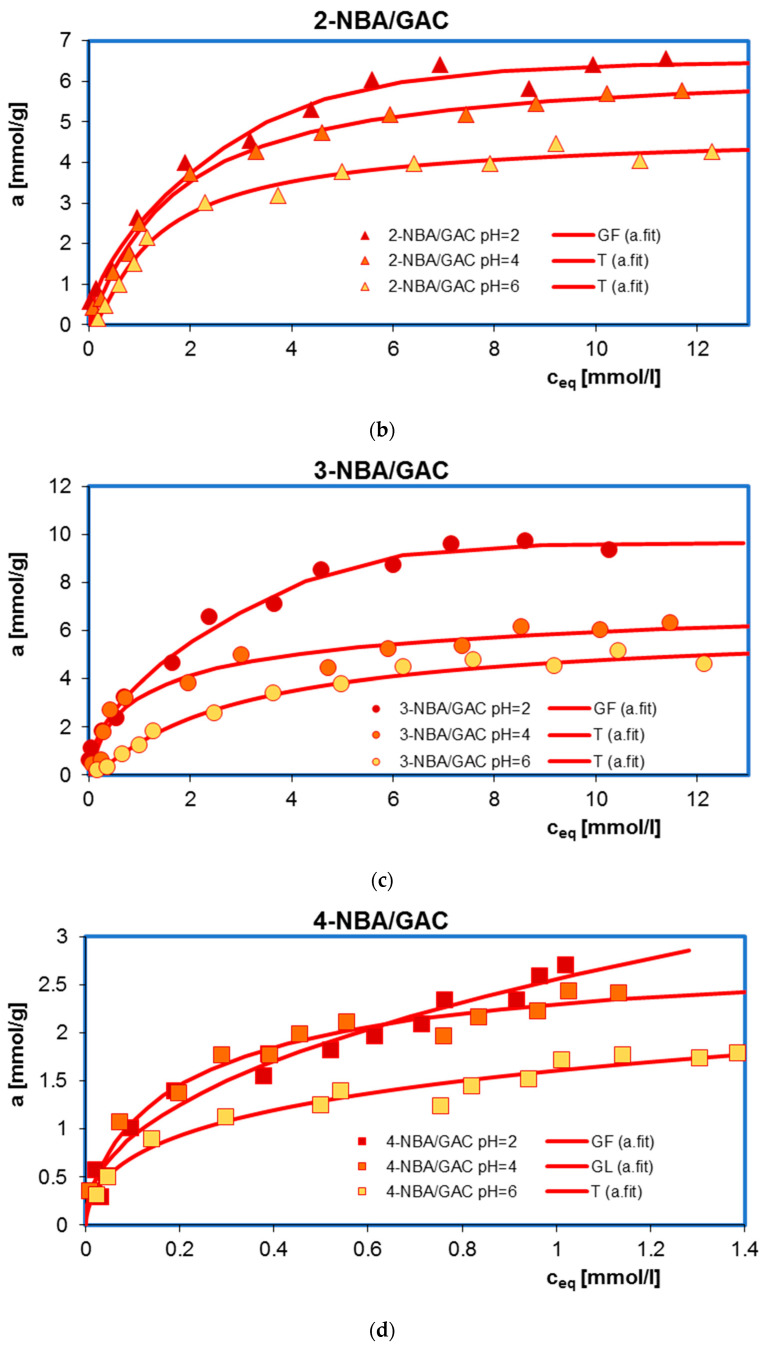

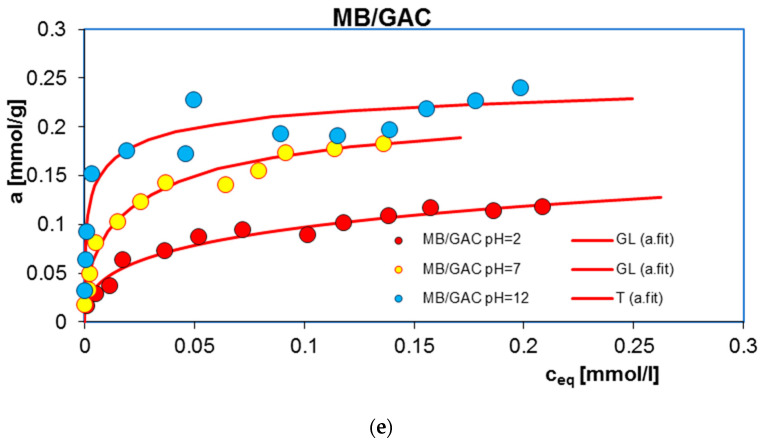
Comparison of adsorption isotherms for BA (**a**), 2-NBA (**b**), 3-NBA (**c**), 4-NBA (**d**), and MB (**e**) on GAC at different solution pH values.

The influence of solution pH on the adsorption equilibrium of benzoic acids, 2-nitrobenzoic acid, 3-nitrobenzoic acid, and 4-nitrobenzoic acid, as well as methylene blue on GAC 1240W activated carbon was examined. Figure 1 presents a comparison of adsorption isotherms in the tested experimental systems.

A clear effect of solution pH on the adsorption efficiency of BA, 2-NBA, 3-NBA, 4-NBA, and MB onto GAC is observed. The adsorption mechanism for individual neutral and ionic forms of adsorbates selected for testing is diverse. At the same time, it should be noted that in the case of all tested experimental systems, π-π dispersion interactions occurred between electrons from the aromatic rings of adsorbates and electrons from the graphene layers of the adsorbent. The remaining interactions resulted from the charge value, both on the surface of the activated carbon and the chemical form of organic substances at the given pH conditions of the solution.

For all acids, a decrease in the amount of adsorption was observed with increasing pH. The obtained maximum adsorption amounts were 11.59 mmol/g, 6.57 mmol/g, 9.38 mmol/g, and 2.70 mmol/g for BA, 2-NBA, 3-NBA, and 4-NBA, respectively. It should be noted that under given conditions of pH = 2, 4, 6, the carbon adsorbent is positively charged (pH_PZC_ = 10.8). Additionally, the pK_a_ for BA, 2-NBA, 3-NBA, and 4-NBA are 4.2, 2.17, 3.45 and 3.44 [3,4,5,6], respectively, which indicates that under strongly acidic pH conditions (pH = 2), virtually all molecules of aromatic organic acids exist in molecular (undissociated) form. Therefore, at pH = 2, there is no electrostatic repulsion in the adsorption system, and as a result, the adsorption is the largest. In these conditions, the adsorption mechanism is mainly based on the occurrence of dispersion forces between the aromatic rings of benzoic, 2-nitrobenzoic, 3-nitrobenzoic, and 4-nitrobenzoic acids and the graphene surfaces of the adsorbent; however, formation of chemical bonds between the functional groups of the adsorbates and the surface groups of the GAC activated carbon is also possible. As the pH increases, due to adsorbate dissociation, in addition to the molecules of aromatic organic acids, their negatively charged ions appear, while the GAC surface still has a positive charge. Therefore, there is an additional electrostatic attraction in the experimental system, which should promote adsorption. As can be seen, in the case of adsorption of BA, 2-NBA, 3-NBA, and 4-NBA, the effect is opposite. This is probably due to the occurrence of competitive adsorption for the active carbon sites between anions originating from the dissociation of adsorbates and hydroxyl anions [29].

In the case of studies on the influence of pH on the adsorption of methylene blue (Figure 1e), an increase in the adsorption with increasing solution pH was observed. Under the tested conditions, activated carbon initially has a positive charge (pH = 2, 7) and then a negative charge (pH = 12). At the same time, the pK_a_ for MB is >12 [7], which means that only in a strongly alkaline environment do the methylene blue molecules appear in solution in the form of cations. In a low pH environment, the tested dye exists in a molecular form, so there are no electrostatic repulsive forces in the adsorption system. Therefore, the adsorption mechanism can be explained based on the occurrence of dispersion forces between the π electrons of the MB aromatic rings and the electrons of the graphene surfaces of activated carbon. In a strongly alkaline environment (pH = 12), the adsorbate takes the form of positive ions and the adsorbent is negatively charged, as a result of which electrostatic attraction forces appear in the adsorption system, which favors adsorption. The received maximum adsorption amount was 0.24 mmol/g.

**Figure 2:**
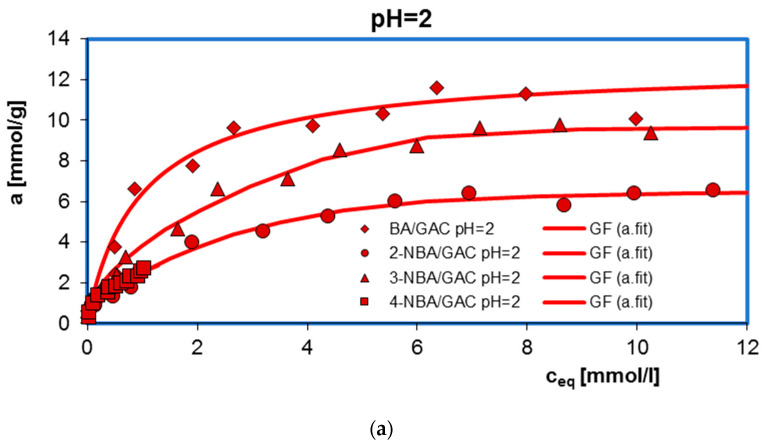

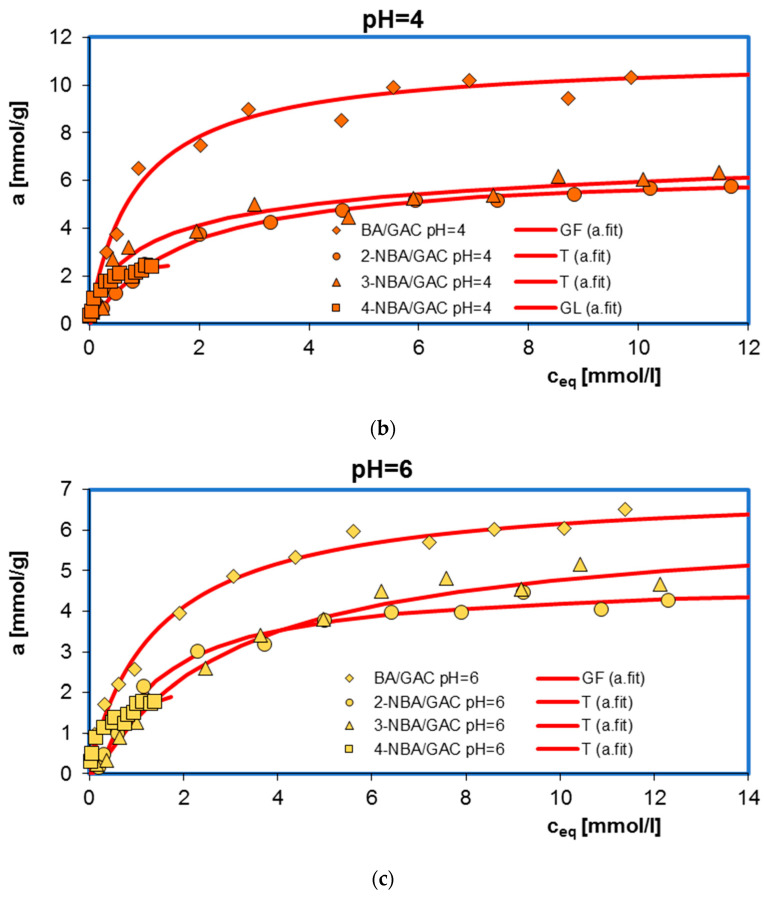
Comparison of adsorption isotherms at different solution pH values of 2 (**a**), 4 (**b**), and 6 (**c**) for BA, 2-NBA, 3-NBA, and 4-NBA on GAC.

Figure 2 additionally shows a comparison of adsorption isotherms for benzoic acid and its nitro derivatives on activated carbon (GAC) under specific pH conditions. As can be seen, the highest adsorption value was recorded for BA (6.52–11.59 mmol/g), a smaller one for 3-NBA (5.16–9.74 mmol/g), then for 2-NBA (4.26–6.57 mmol/g), and the smallest for 4-NBA (1.77–2.70 mmol/g). This effect can be explained based on differences in the water solubility of the tested adsorbates. The solubility is 2.9 g/L, 6.8 g/L, 3 g/L, and 0.42 g/L for BA, 2-NBA, 3-NBA, and 4-NBA, respectively. Generally, substances that are less soluble in water have a greater affinity for the hydrophobic surface of activated carbon [30]. In the presented research, the above rule does not apply only in the case of the adsorption of 4-nitrobenzoic acid on GAC. At the same time, taking into account that 4-NBA has up to 16 times lower solubility compared to other adsorbates, the initial concentrations of 4-nitrobenzoic acid solutions were also much lower.

The adsorption equilibrium experimental data were studied using the generalized Langmuir (GL) equation, and its parameters are listed in Table 1. It should be emphasized that adsorption in micro- and mesoporous systems can most often be described using a general integral equation assuming energetic heterogeneity. When we take into account the limited range of concentrations and the accuracy of the available data, equations assuming a certain nature of the adsorption energy distribution are sufficient to describe them. Due to their high generality and flexibility, the GL or one of its specific forms, Langmuir–Freundlich (LF), the generalized Freundlich (GF), Tóth (T), or even the Langmuir (L) isotherm, seem to be very good isotherms for analysis of adsorption data.

As can be seen, for the three tested adsorption systems (4-NBA/GAC pH = 4; MB/GAC pH = 2; and MB/GAC pH = 7) the inhomogeneity parameters m and n were lower than one, so the full form of the GL equation was used here. In the case of the BA/GAC and 2-, 3-, and 4-NBA/GAC systems at pH = 2, the heterogeneity parameter n is equal to one; therefore, a simplified form of the generalized Langmuir isotherm was used for their analysis—the generalized Freundlich (GF) equations. For the remaining experimental series, the parameter m is equal to one, so the Tóth isotherm equation is the best for their analysis. From a practical point of view, this means that medium heterogeneity effects were observed in the system. The fitted adsorption values were in the ranges of 6.86–11.68 mmol/g, 4.82–6.59 mmol/g, 5.47–9.84 mmol/g, 2.03–3.14 mmol/g, and 0.17–0.29 mmol/g for BA, 2-NBA, 3-NBA, 4-NBA, and MB. These values are very close to the values determined experimentally. The quality of the fit is very good, as evidenced by the high values of the coefficients of determination (R^2^ = 0.932–0.995) and low values of standard deviations (SD(a) = 0.012–0.096).

### 2.2. Adsorption Kinetics

**Figure 3:**
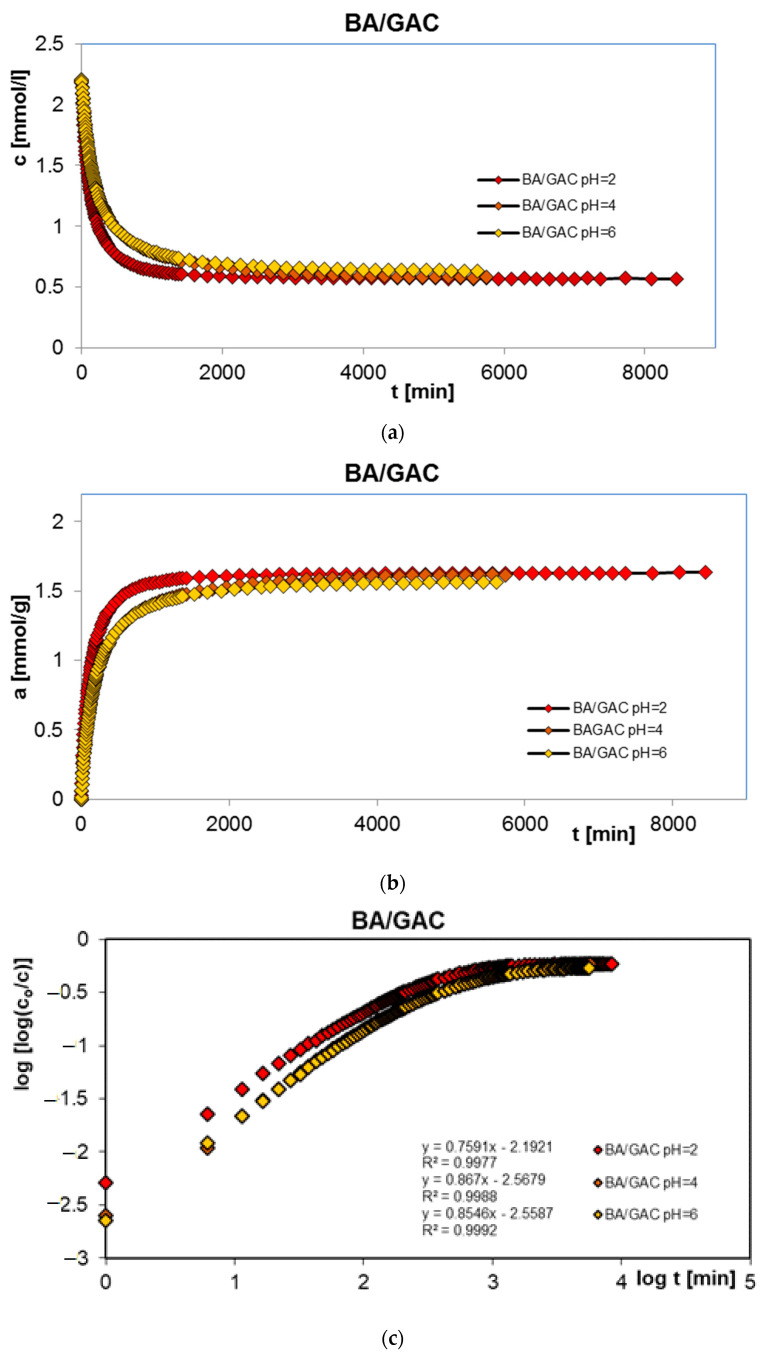
Comparison of the kinetics of adsorption of benzoic acid (BA) on GAC activated carbon at different solution pH values presented in the profile of concentration changes over time (**a**), relative adsorption over time (**b**), and in Bangham’s linear coordinates (**c**).

**Figure 4:**
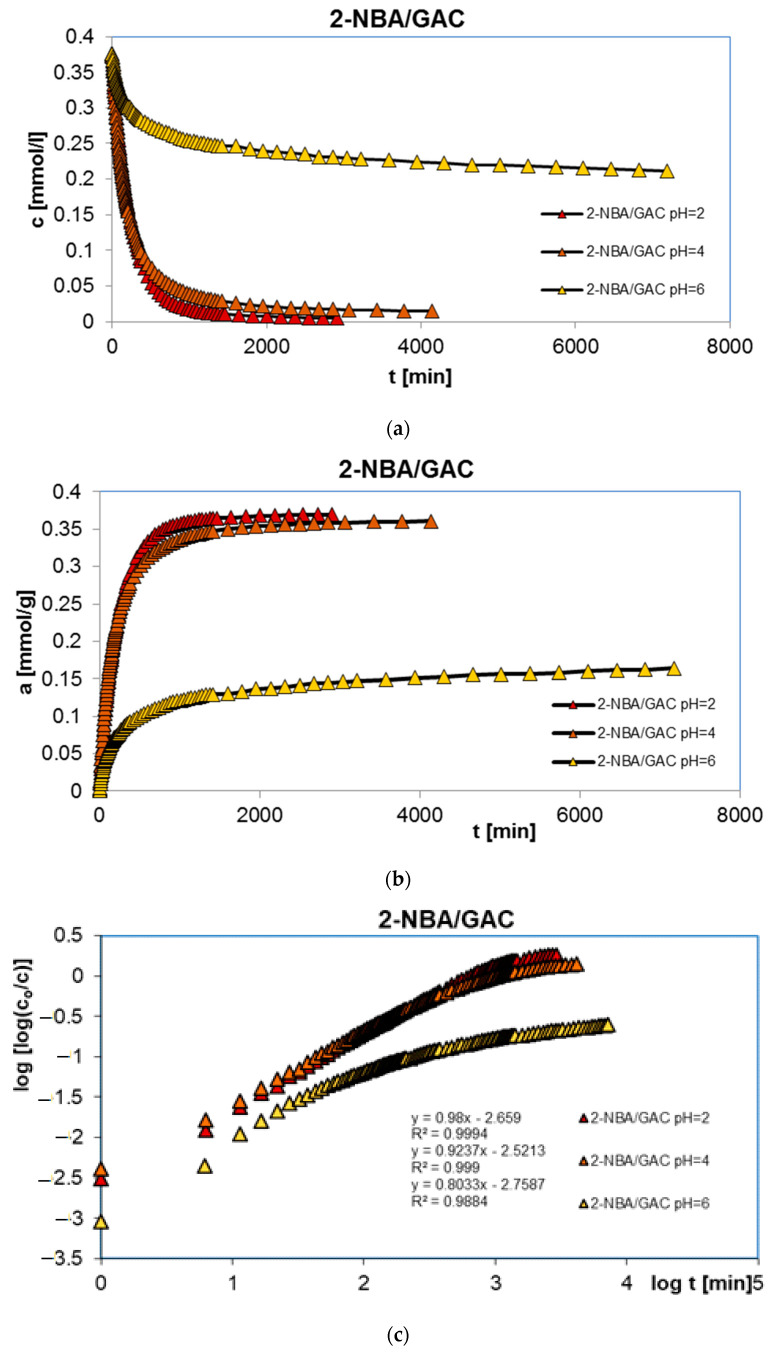
Comparison of the kinetics of adsorption of 2-nitrobenzoic acid (2-NBA) on GAC activated carbon at different solution pH values presented in the profile of concentration changes over time (**a**), relative adsorption over time (**b**), and in Bangham’s linear coordinates (**c**).

**Figure 5:**
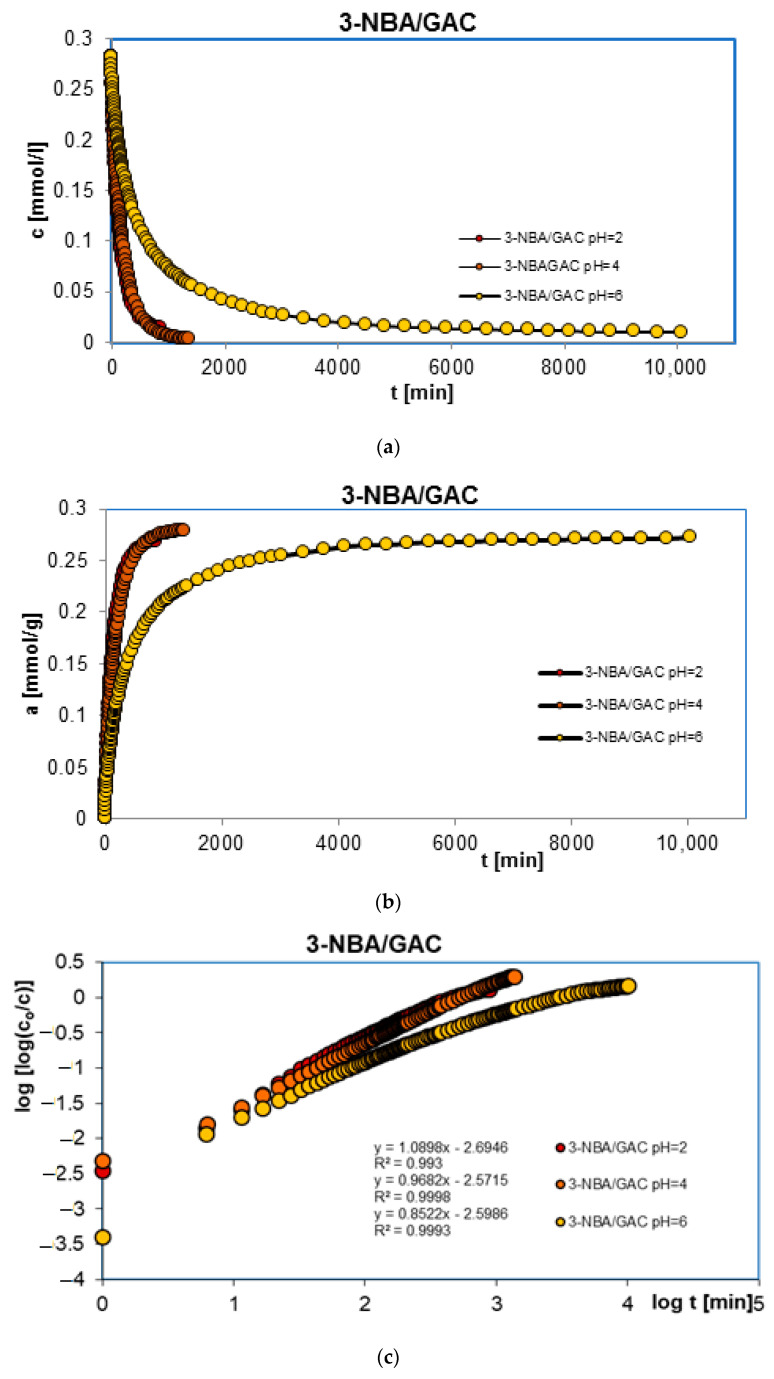
Comparison of the kinetics of adsorption of 3-nitrobenzoic acid (3-NBA) on GAC activated at different solution pH values presented in the profile of concentration changes over time (**a**), relative adsorption over time (**b**), and in Bangham’s linear coordinates (**c**).

**Figure 6:**
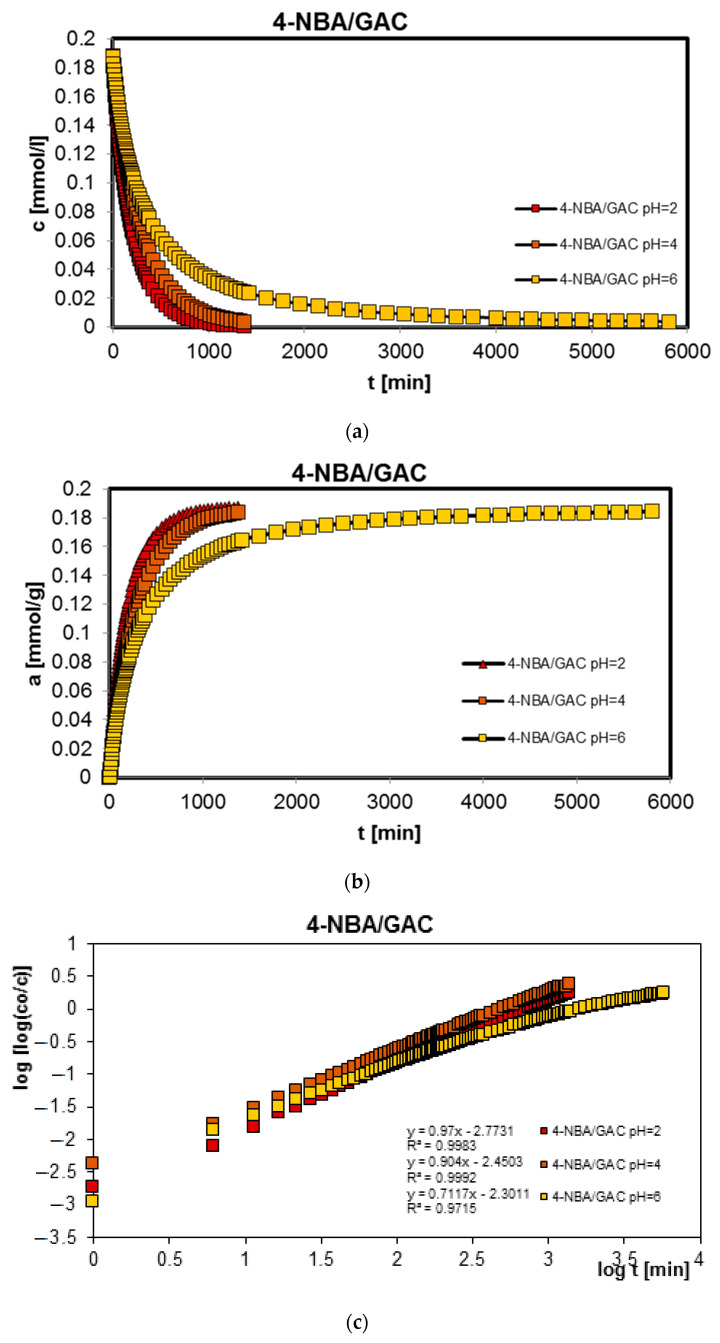
Comparison of the kinetics of adsorption of 4-nitrobenzoic acid (4-NBA) on GAC activated carbon at a constant initial adsorbate concentration and variable adsorbent mass presented in the profile of concentration changes over time (**a**), relative adsorption over time (**b**), and in Bangham’s linear coordinates (**c**).

**Figure 7:**
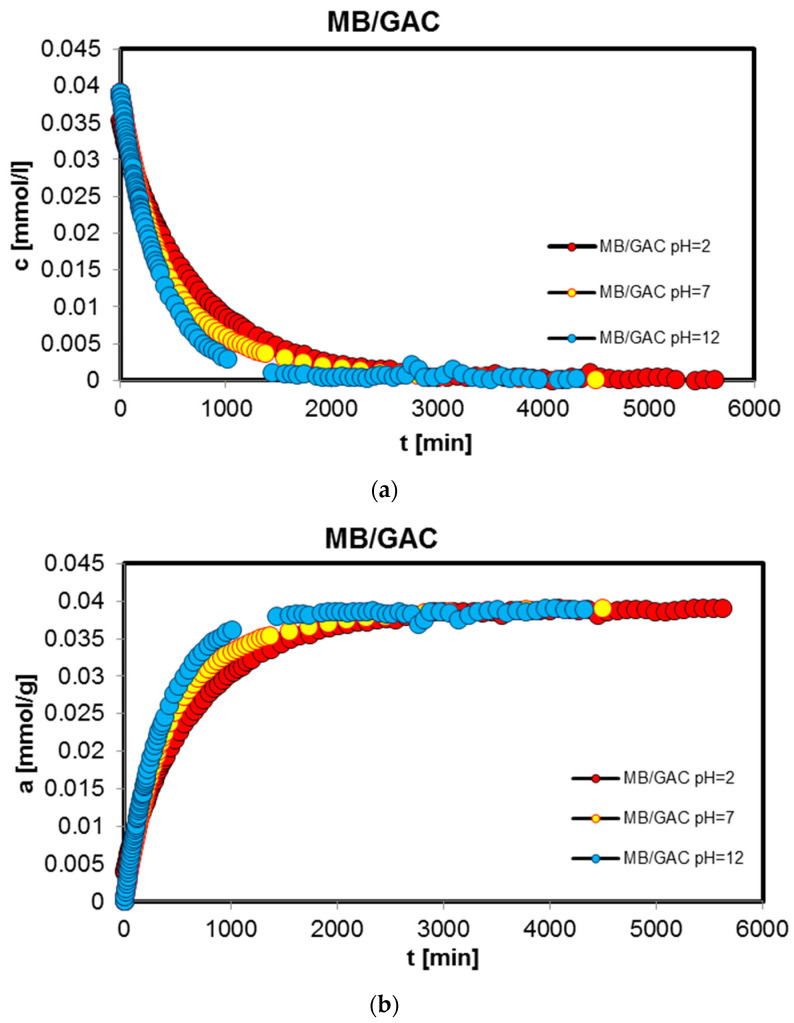

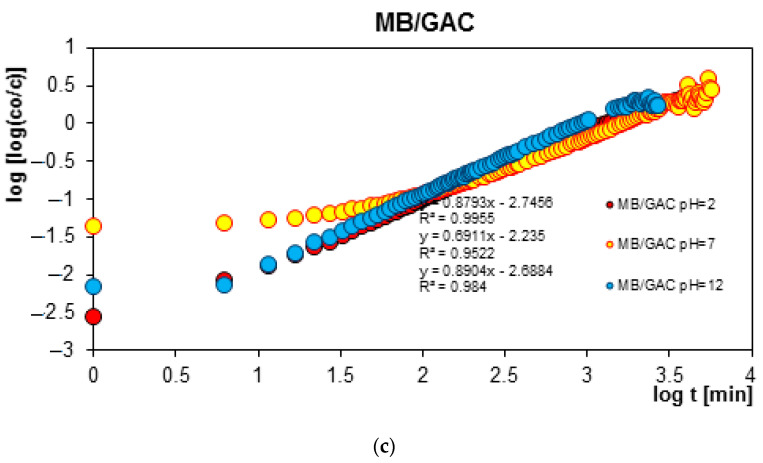
Comparison of the kinetics of adsorption of methylene blue (MB) on GAC activated carbon at different solution pH values presented in the profile of concentration changes over time (**a**), relative adsorption over time (**b**), and in Bangham’s linear coordinates (**c**).

In order to complete the studies on the adsorption of benzoic, 2-nitrobenzoic, 3-nitrobenzoic, 4-nitrobenzoic, and methylene blue acids on activated carbon (GAC) at different solution pH values, adsorption kinetics tests were performed. Similarly, the research was carried out at pH 2, 4, and 6 for organic acids and 2, 7, and 12 for the dye. Figure 3, Figure 4, Figure 5, Figure 6 and Figure 7 show the concentration ((a) in Figure 3, Figure 4, Figure 5, Figure 6 and Figure 7), adsorption ((b) in Figure 3, Figure 4, Figure 5, Figure 6 and Figure 7), profiles in time for BA (Figure 3), 2-NBA (Figure 4), 3-NBA (Figure 5), 4-NBA (Figure 6), and MB (Figure 7) on GAC, and the linear Bangham relationships ((c) in Figure 3, Figure 4, Figure 5, Figure 6 and Figure 7).

As can be seen, in the case of adsorption kinetic studies, similar trends were observed as for the adsorption equilibrium data. A higher adsorption rate was reported under strongly acidic pH conditions for all aromatic organic acids and under strongly basic pH conditions for methylene blue.

The analysis of adsorption kinetics data in the tested experimental systems began with their comparison in linear Bangham coordinates [31]. As can be seen, these graphs are almost linear, and their slopes are within the range of 0.76–0.87, 0.80–0.98, 0.85–1.09, 0.71–0.97, and 0.88–0.95 for BA/GAC, 2-NBA/GAC, 3-NBA/GAC, 4-NBA/GAC, and MB/GAC. Such slope values indicate that the adsorption rate in the tested research systems cannot be described using the intraparticle diffusion model (IDM). It should be mentioned that for the pure IDM model the slopes are equal to 0.5.

Taking into account the preliminary analysis of the adsorption rate data in the tested experimental systems using Bangham’s linear relations, it was shown that diffusion (in the tested systems) was not a factor limiting the adsorption kinetics. Therefore, simple adsorption kinetic equations were used in further considerations, including the first-order, the second-order, the 1,2-mixed-order equations, as well as their fractal equivalents and the multi-exponential equation. The relative standard deviations for FOE, SOE, MOE, f-FOE, f-SOE, f-MOE, and m-exp are listed in Table 2. As can be seen, for all experimental systems, the best fit quality was obtained using the multi-exponential equation (SD(c)/c_o_ = 0.076–0.437%), whose parameters are collected in Table 3. Satisfactory quality of fitting was also achieved using a 1,2-order mixed equation (SD(c)/c_o_ = 0.089–1.801%), the parameters of which are listed in Table 4. 

It should be added here that in the case of adsorption kinetics, in addition to the factors that influence the balance of this process, it is also necessary to take into account diffusion, which depends on the shape and size of the adsorbent grain, as well as the nature and size of its pores, the mixing speed, and temperature. Due to the complicated nature of these relationships, they usually cannot be fully described by simplified models (Langmuir and intragranular diffusion). Therefore, empirical or semi-empirical equations are often used to correctly describe the behavior of the system in practice.

Based on the analysis of the parameter values presented in Table 3, it can be concluded that adsorption in the tested experimental systems is a complex process whose kinetics can be described using two or three terms of the m-exp equation. Additionally, it was observed that the values of half-times t_1/2_ increase with increasing pH for systems with aromatic organic acids and are 96.7–257.8 min, 144–310 min, 99.2–311.9 min, and 133.5–249.3 min, respectively, for BA/GAC, 2-NBA/GAC, 3-NBA/GAC, and 4-NBA/GAC. For MB/GAC, the opposite effect was observed; the values of half-times increased with a decrease in the pH of the solution and ranged from 260.2 to 395.5 min. The quality of fitting the kinetics of the tested adsorbates using the m-exp equation is very good (1 − R^2^ within the range of 1.1 × 10^−5^ to 9.6 × 10^−3^, and relative standard deviations of 0.076–0.437%).

The analysis of the data from Table 4 showed that the kinetics of benzoic acid adsorption is close to second-order kinetics (f_2_ = 0.884–0.998), while the kinetics of methylene blue is closer to first-order kinetics (f_2_ = 0.038–0.423). In the case of nitrobenzoic acids, a significant contribution of second-order kinetics was observed at higher pH values (2-NBA: f_2 pH=2_ = 0.335, f_2 pH=4_ = 0.722, and f_2 pH=6_ = 0.997; 3-NBA: f_2 pH=2_ = 0.302, f_2 pH=4_ = 0.302, and f_2 pH=6_ = 0.997; and 4-NBA: f_2 pH=2_ = 0.174, f_2 pH=4_ = 0.295, and f_2 pH=6_ = 0.889). The quality of the fit of the kinetics of the tested substances is very good or good (1 − R^2^ within the range of 1.1 × 10^−5^ to 9.6 × 10^−3^, and relative standard deviations of 0.089–1.801%). 

It should also be noted that in the case of 2-, 3-, and 4-nitrobenzoic acids and methylene blue, a loss of adsorbate from the solution close to 100% (u_eq_ ≈ 1) was observed. However, for benzoic acid (with a much higher concentration) the u_eq_ parameter is ≈0.7. According to Langmuir’s theory, for an ideal adsorption system, the product of relative adsorption and loss $\theta_{eq}u_{eq}$ is equal to the relative contribution of second-order kinetics; however, for 2-, 3-, 4-NBA and MB, the amount of adsorption a_eq_ (and with similar adsorption capacity—also the relative adsorption $\theta_{eq}$) is several times lower than for BA and hence the f_2_ values are smaller.

### 2.3. Thermal Analysis

**Figure 8:**
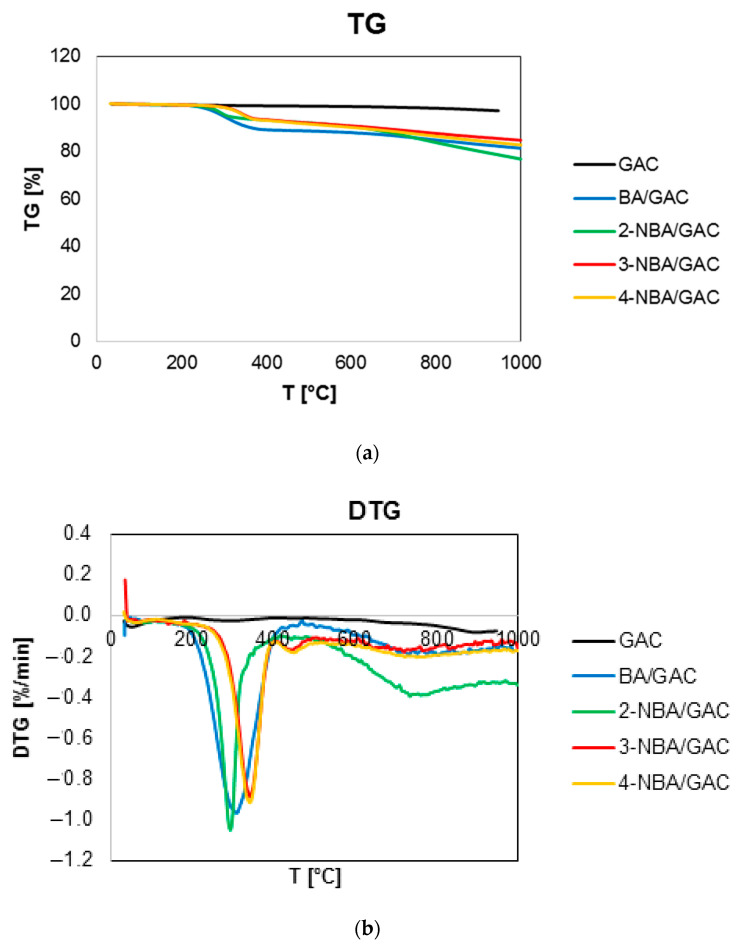

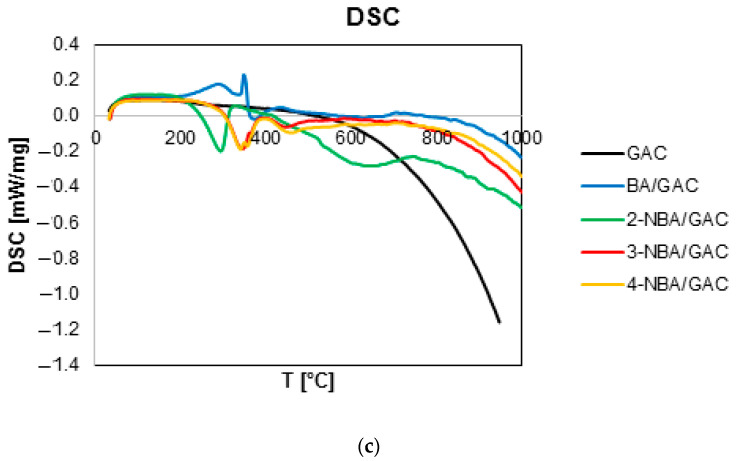
Comparison of TG (**a**), DTG (**b**), and DSC (**c**) curves GAC activated carbon before and after BA, 2-, 3-, and 4-NBA adsorption.

In order to determine and compare the strength of interactions between the adsorbent and benzoic acid and its nitro derivatives, the measurements by using thermal analysis were carried out in an inert gas atmosphere for BA, 2-, 3-, and 4-NBA/GAC, and for comparative purposes for pure activated carbon GAC. Figure 8 presents a comparison of TG, DTG, and DSC curves for activated carbon before and after the adsorption of aromatic organic acids. Additionally, Figures S1–S3 show the MS profiles of the main gaseous degradation products of the above-mentioned samples. Moreover, Table 5 presents data determined based on the analysis of TG, DTG, and DSC curves. Finally, Table 6 compares the *m*/*z* values found in the mass spectrum of the GAC material before and after the adsorption of BA, 2-, 3-, and 4-NBA.

It was observed that the decomposition of GAC activated carbon in the nitrogen atmosphere basically occurs in two stages with a total mass loss of 3.03%. The first one, in the temperature range from 30 to 180 °C and with a mass loss of 0.48%, corresponds to the endothermic process of desorption of physically adsorbed water. This is confirmed by the occurrence of maximum peaks in the mass spectrum *m*/*z* = 17 (97 °C) for OH and *m*/*z* = 18 (90 °C) for H_2_O. The second stage, in the temperature range from 180 to 1000 °C, corresponds to the exothermic removal of surface functional groups of activated carbon and its partial defragmentation (*m*/*z* = 17 for OH (271 °C); *m*/*z* = 18 for H_2_O (289 °C); *m*/*z* = 44 for CO_2_ (283 and 720 °C); and *m*/*z* = 78 for C_6_H_6_ (277, 495, 636, and 887 °C)). It should also be noted that the *m*/*z* = 78 spectrum from C_6_H_6_ is most visible at 277 °C. Additionally, this MS profile is the least intense of all registered MS profiles, as evidenced by the lowest values of ion current, which was of the order of 10^−13^ A.

The decomposition of carbons loaded with adsorbates occurs with a total mass loss of 18.77%, 23.39%, 15.40%, and 17.38% for BA/GAC, 2-NBA/GAC, 3-NBA/GAC, and 4-NBA/GAC, respectively. There are three stages observed during these processes. The first one, in the temperature range of 30–180 °C, is responsible for endothermically removing physically bound water (visible peaks in the mass spectrum for *m*/*z* = 17 (OH) and *m*/*z* = 18 (H_2_O). Then, in the range of 180–500 °C, defragmentation and removal of the adsorbate from the adsorbent surface take place. In the case of BA/GAC, this process is endothermic, during which the occurrence of numerous species in the mass spectrum can be distinguished, including the following: OH (*m*/*z* = 17; 279 and 369 °C), H_2_O (*m*/*z* = 18; 294 and 359 °C), C_3_H_3_^+^ (*m*/*z* = 39; 309 °C), CO_2_ (*m*/*z* = 44; 369 °C), COOH^+^ (*m*/*z* = 45; 364 °C), C_4_H_2_^+^ (*m*/*z* = 50; 299 °C), C_4_H_3_^+^ (*m*/*z* = 51; 304 °C), C_5_H_5_^+^ (*m*/*z* = 65; 289 °C), C_6_H_3_^+^ (*m*/*z* = 75; 294 °C), C_6_H_6_ (*m*/*z* = 78; 304 °C), C_6_H_5_C=O^+^ (*m*/*z* = 105; 304 °C), and C_6_H_5_COOH (*m*/*z* = 122; 304 °C). The most intense signals were obtained for *m*/*z* = 17, 18, 44, and 51, and their ion currents were of the order of 10^−10^ A. The least visible signals came from *m*/*z* = 45, 65, and 122 (ion current of 10^−13^ A) However, for the 2-, 3-, and 4-NBA/GAC systems, the second stage of decomposition is exothermic, during which the presence of peaks in the mass spectrum from the following species can be observed: OH (*m*/*z* = 17; 304 °C (2-NBA), 365 °C (3-NBA), and 366 °C (4-NBA)), H_2_O (*m*/*z* = 18; 305 °C (2-NBA), 370 °C (3-NBA), and 371 °C (4-NBA)), NO (*m*/*z* = 30; 305 °C (2-NBA), 365 and 450 °C (3-NBA), and 366 and 466 °C (4-NBA)), C_3_H_3_^+^ (*m*/*z* = 39; 199 and 304 °C (2-NBA), 190 and 360 °C (3-NBA), and 181 and 356 °C (4-NBA)), CO_2_ (*m*/*z* = 44; 300 and 759 °C (2-NBA), 360 °C (3-NBA), and 361 °C (4-NBA)), COOH^+^ (*m*/*z* = 45; 300 and 329 °C (2-NBA), 355 °C (3-NBA), and 356 °C (4-NBA)), NO_2_ (*m*/*z* = 46; 305 and 764 °C (2-NBA), 345 °C (3-NBA), and 351 °C (4-NBA)), C_4_H_2_^+^ (*m*/*z* = 50; 304 °C (2-NBA), 365 °C (3-NBA), and 361 °C (4-NBA)), C_4_H_3_^+^ (*m*/*z* = 51; 310 °C (2-NBA), 360 °C (3-NBA), and 361 °C (4-NBA)), C_5_H_5_^+^ (*m*/*z* = 65; 304 °C (2-NBA), 360 °C (3-NBA), and 361 °C (4-NBA)), C_6_H_3_^+^ (*m*/*z* = 75; 300 °C (2-NBA), 350 °C (3-NBA), and 361 °C (4-NBA)), and C_6_H_6_ (*m*/*z* = 78; 310 °C (2-NBA), 351 °C (3-NBA), and 356 °C (4-NBA)). As can be seen, the MS profiles for all *m*/*z* tests recorded in this part were the most intense for the 2-NBA/GAC sample. Ion currents for this system were several times higher than for systems with 3-NBA and 4-NBA. Moreover, the highest values of ion currents (of the order of 10^−8^ A) were recorded for *m*/*z* = 17, 18, and 44. Additionally, the recorded peak maximum in the MS profiles for individual *m*/*z* are shifted towards higher temperatures for samples with 3-nitrobenzoic acid and 4-nitrobenzoic acid.

The last stage corresponds to the final decomposition of aromatic organic acids and partial defragmentation of the GAC activated carbon.

It should also be noted that the decomposition of the 2-NBA/GAC sample occurs already at 294.4 °C (DTG), while for 3-NBA/GAC and 4-NBA/GAC it occurs at 339.9 °C and 341.3 °C (DTG), respectively. Moreover, BA/GAC decomposition occurs at 299.4 °C (DTG). This indicates stronger interactions of 3- and 4-nitrobenzoic acids with GAC activated carbon in relation to other adsorbates. The weakest interaction in the 2-NBA/GAC system may result from the possibility of an intramolecular bond forming between the functional groups in the ortho position of 2-nitrobenzoic acid.

### 2.4. X-ray Photoelectron Spectroscopy (XPS)

**Figure 9:**
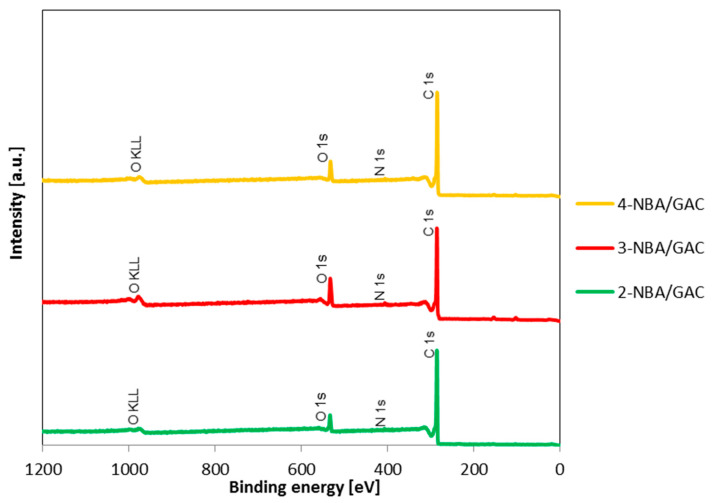
XPS survey spectra of 2-NBA/GAC, 3-NBA/GAC, and 4-NBA/GAC.

**Figure 10:**
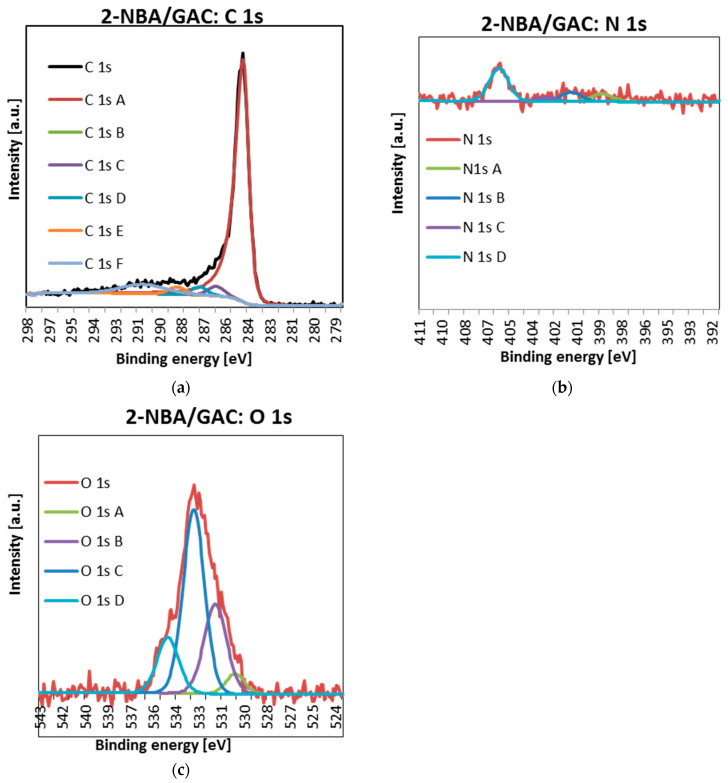
Deconvoluted C 1s (**a**), O 1s (**b**), and N 1s (**c**) high-resolution core-level XPS spectra 2-NBA/GAC.

**Figure 11:**
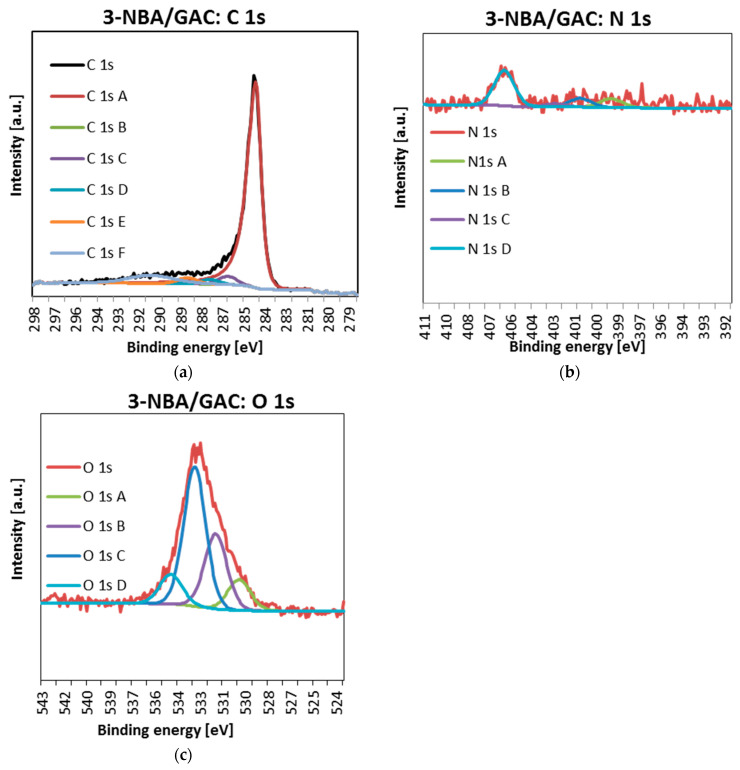
Deconvoluted C 1s (**a**), O 1s (**b**), and N 1s (**c**) high-resolution core-level XPS spectra 3-NBA/GAC.

**Figure 12:**
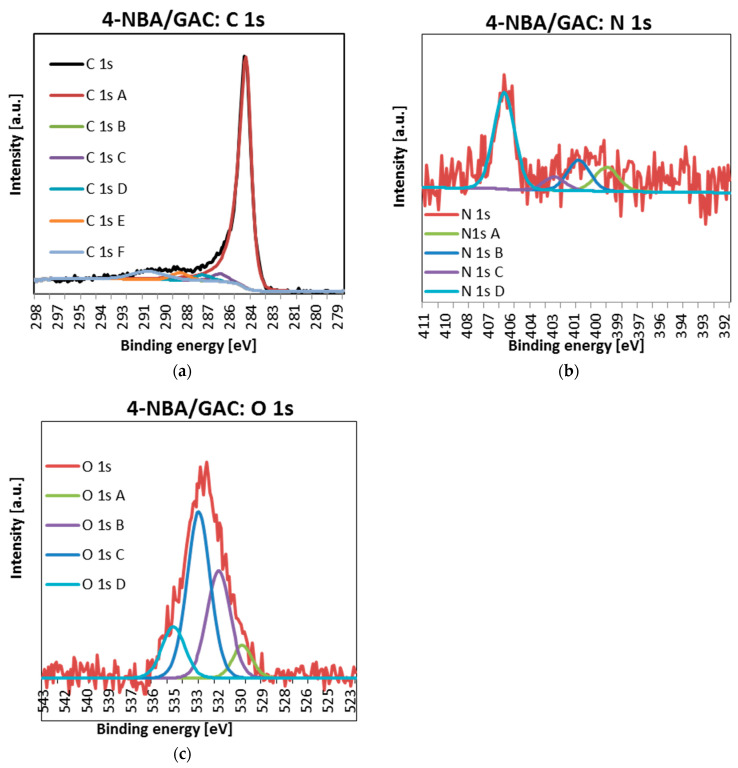
Deconvoluted C 1s (**a**), O 1s (**b**), and N 1s (**c**) high-resolution core-level XPS spectra 4-NBA/GAC.

In order to determine the elemental composition and chemical bonds occurring in the studied materials after the adsorption of nitrobenzoic acids on activated carbon (GAC), XPS measurements were performed. The received data are shown in Figure 9, Figure 10, Figure 11 and Figure 12 and in Table 7 and Table 8. Figure 9 shows the comparison of XPS survey spectra for 2-NBA/GAC, 3-NBA/GAC, and 4-NBA/GAC. As can be seen, three bands were revealed at binding energies (BE) of 284.8 eV, ~406 eV, and ~533 eV, originating from carbon C 1s, nitrogen N 1s, and oxygen O 1s, respectively. The most intense peaks come from carbon C 1s, and the least visible ones come from nitrogen N 1s. The atomic concentrations of the examined elements were also determined: C 1s—92.5 at.%, O s1—6.6 at.%, and N 1s—0.8 at.% for 2-NBA/GAC; C 1s—86.3 at.%, O s1—12.2 at.%, and N 1s—1.5 at.% for 2-NBA/GAC; and C 1s—91.3 at.%, O s1—7.5 at.%, and N 1s—1.2 at.% for 4-NBA/GAC (Table 7). 

In a further part of the research, the detailed spectra of carbon C1s, nitrogen N1s, and oxygen O1s were obtained using the XPS technique. The obtained data are presented in Figure 10, Figure 11 and Figure 12 and Table 8.

As can be seen, for all materials, the carbon lines are divided into five singlet spectral lines (Figure 10a, Figure 11a and Figure 12a, Table 8). The first one, with a binding energy of 284.5 eV, comes from a carbon atom bonded with a double bond to the second carbon atom C=C. It should be added that this is the most intense peak among all C1s carbon peaks. The next one, at BE = 285 eV, corresponds to a single C-C bond between carbon atoms and a carbon–hydrogen bond C-H. The next signal, at a binding energy of 286.2 eV, indicates the presence of carbon bonded to the oxygen of the hydroxyl group C-OH and the single bond of oxygen to two carbon atoms C-O-C. The fourth signal, at BE = 287.3 ev, corresponds to the double bond between the carbon and oxygen atoms C=O. The last one, with a binding energy of 288.7 eV, suggests the presence of a carbon from the COOH carboxyl group [32,33].

Analysis of Figure 10b, Figure 11b and Figure 12b and Table 8 shows that the nitrogen lines are divided into four singlet spectral lines. The lines at the binding energy of ~399 and ~401 eV correspond to nitrogen bound to the organic matrix. The next signal, at BE = 402.7 eV, indicates the presence of nitrogen from nitrous oxide. The last line, with a binding energy of ~406 eV, corresponds to the presence of nitrogen in the nitro group [32,33]. The last peak, indicating the presence of the NO_2_ group, is the most visible of all the peaks regarding the presence of nitrogen N 1s.

Finally, examination of Figure 10c, Figure 11c and Figure 12c and Table 8 indicates that oxygen lines are distributed into four singlet spectral lines. The first one, with a binding energy of ~530 eV, corresponds to an oxygen from the quinone group. The next line, with BE ~ 531.6 eV, suggests the presence of oxygen bound by a double bond to carbon O=C or in the O=C-O-R group. The next line, with a binding energy of ~533 eV, is associated with the presence of oxygen from the hydroxyl group connected to the aromatic ring. The above peak, indicating the presence of an Ar-OH connection, is the most intense of all peaks related to the presence of oxygen O 1s. The last line, at BE ~ 534.6 eV, corresponds to oxygen connected by single bonds to carbon and an alkyl substituent O=C-O-R [32,33].

It is also worth paying special attention to the percentage of individual forms of nitrogen. As you can see, the nitrogen content bound to the organic matrix ranges from 30.2 to 33.4%. Therefore, this suggests that the formation of bonds between the surface groups of GAC activated carbon and the functional groups of nitrobenzoic acids occurs mainly through the carboxyl group. 

## 3. Discussion

This work presents the results of studies on the equilibrium and kinetics of adsorption of benzoic, 2-nitrobenzoic, 3-nitrobenzoic, 4-nitrobenzoic, and methylene blue acids on granular activated carbon (GAC). The developed research methodology enabled experiments to be conducted under strictly controlled temperature and mixing conditions. Moreover, the kinetic data contained up to 120 experimental points. Analysis of sorption data using many models and equations of equilibrium and adsorption kinetics allowed for a precise estimation of the adsorption mechanism for selected aromatic organic compounds on activated carbon (GAC). Additionally, data from thermal analysis in a nitrogen atmosphere made it possible to determine the strength of interactions of benzoic acid and its nitro derivatives with the adsorbent surface. Moreover, data from XPS measurements allowed the indication of the possibility of creating a chemical bond between the surface groups of activated carbon (GAC) and nitrobenzoic acid molecules. There are works in the literature on the subject, examining the influence of pH on the adsorption of aromatic organic compounds on activated carbon. At the same time, there is a lack of comprehensive studies of the equilibrium and adsorption kinetics for benzoic acid and all its nitro derivatives.

Derylo-Marczewska and Marczewski [34] studied the adsorption equilibrium of benzoic acid on RIAA, RIB, and RIC activated carbons at pH values close to 2 and 9 at 25 °C. Later, Derylo-Marczewska and Marczewski [35] extended the adsorption equilibrium studies for 2-nitrobenzoic acid, 3-nitrobenzoic acid, and 4-nitrobenzoic acid on RIB activated carbon at pH = 2.2 at 25 °C. Ayranci et al. [36] studied the adsorption of benzoic acid on carbon cloth, with equilibrium at pH 2.0, 3.7, and 11.0 and adsorption kinetics at pH 2.0, 3.7, 5.3, 9.1, and 11.0 at 25 °C. Xin et al. [37] examined the efficiency of removing benzoic acid from an aqueous solution using three kinds of modified bentonites in the pH range 2–8. Additionally, at pH = 3.5 (optimal conditions), the adsorption kinetics was also studied. 

## 4. Materials and Methods

### 4.1. Chemicals

The following aromatic organic compounds were used as adsorbates in the research: benzoic acid (BA; Merck, Darmstadt, Germany), 2-nitrobenzoic acid (2-NBA; Aldrich, Steinheim, Germany), 3-nitrobenzoic acid (3-NBA; Merck, Schuchardt, Germany), 4-nitrobenzoic acid (4-NBA; Merck, Schuchardt, Germany), and methylene blue (MB; Sigma-Aldrich, St. Louis, MO, USA). Their selected physicochemical properties and structure are presented in Table 9.

Commercially available activated carbon GAC 1240W (GAC) was used as an adsorbent for the tests, which was selected from Norit (The Netherlands) after their offer. A detailed description of its structural and textural, morphological, and acid–base properties is presented in [29,30]. This material was characterized by a highly developed structure and irregular grain shape. The basic textural parameters are as follows: specific surface area S_BET_ = 900 m^2^/g, external surface area S_EXT_ = 523 m^2^/g, total pore volume V_t_ = 0.52 cm^3^/g, micropore volume V_mic_ = 0.20 cm^3^/g, and average hydraulic pore size d_h_ = 2.31 nm. This activated carbon also has alkaline properties; its pH_PZC_ was 10.8 [29,30].

### 4.2. Methods

#### 4.2.1. Adsorption Equilibrium

**Figure 13:**
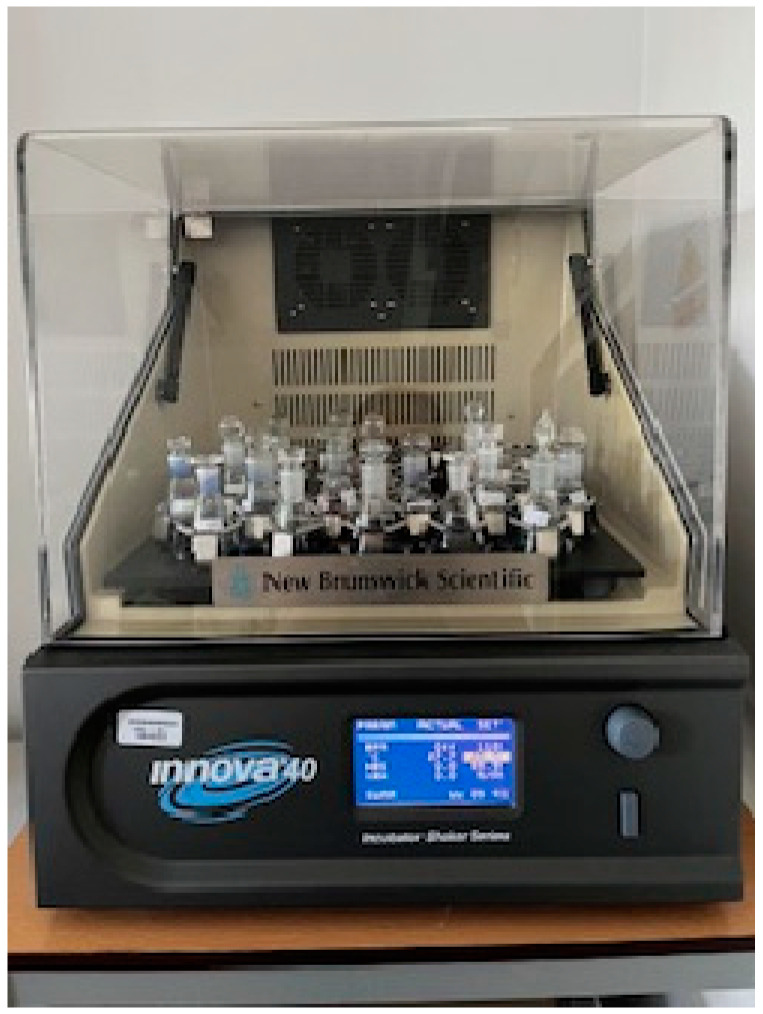
Incubator shaker Innova 40 (New Brunswick).

Equilibrium adsorption measurements of benzoic, 2-, 3-, and 4-nitrobenzoic acids and methylene blue on granular activated carbon (GAC) were performed at various pH conditions. The pH values were adjusted using small amounts of hydrochloric acid and sodium base. Adsorption measurements of BA, 2-NBA, 3-NBA, and 4-NBA were carried out at pH 2, 4, and 6. However, tests for methylene blue were carried out at pH 2, 7, and 12. Adsorption systems were prepared in Erlenmayer flasks at a temperature of 25 °C, and shaken in a thermostated incubator for 7 days with a stirring speed of 110 rpm (Figure 13). All spectrophotometric measurements (Cary 4000 UV–vis spectrophotometer; Varian, Australia) were carried out with the registration of spectra in the range of 200–800 nm. The absorption maximum was observed at the following wavelengths: 273 (BA), 265 (2- and 4-NBA), 261 (3-NBA), and 664 nm (MB).

The generalized Langmuir adsorption isotherm (GL; also called the Marczewski–Jaroniec (M-J) isotherm) equation was used to analyze the adsorption equilibrium data. It describes well the adsorption from solutions on solids characterized by energetic heterogeneity. The GL isotherm equation is as following:

(1) $$\theta ={\left[\frac{{\left(\bar{K}\cdot c_{eq}\right)}^{n}}{{1+\left(\bar{K}\cdot c_{eq}\right)}^{n}}\right]}^{\frac{m}{n}}$$

where the following are defined: Ɵ—surface coverage, Ɵ = a/a_m_; m and n—heterogeneity parameters; K—the adsorption equilibrium constant; and c_eq_—equilibrium concentration. In some cases, the generalized Langmuir isotherm simplifies to the generalized Freundlich (GF; n = 1), Langmuir–Freundlich (LF; m = n), Tóth (T; m = 1), or Langmuir (L; m = n = 1) equations [38,39].

#### 4.2.2. Adsorption Kinetics

Adsorption kinetics of benzoic, 2-, 3-, and 4-nitrobenzoic acids, as well as methylene blue, on activated carbon (GAC) was measured under various pH conditions. The pH was adjusted with small amounts of acid and base. Measurements of the adsorption of benzoic acid and its nitro derivatives were carried out at pH values equal to 2, 4, and 6. However, the sorption tests of methylene blue were carried out at pH values equal to 2, 7, and 12. The concentrations of the adsorbate solutions were equal to 2.2, 0.376, 0.282, 0.188, and 0.039 mmol/L for BA, 2-NBA, 3-NBA, 4-NBA, and MB, respectively, and the ratio of adsorbent mass to adsorbate volume was 1 g/L. Measurements were carried out in a double-walled thermostated vessel at a temperature of 25 °C. The solution was mixed using a digitally controlled mechanical mixer at a speed of 110 rpm. All spectrophotometric measurements (Cary 100 UV–vis spectrophotometer; Varian, Australia) were carried out with the registration of spectra in the range of 200–800 nm. The absorption maximum was observed at the following wavelengths: 273 (BA), 265 (2- and 4-NBA), 261 (3-NBA), and 664 nm (MB). 

Based on the obtained experimental data, changes in the concentration of the tested adsorbates over time were determined, and the obtained relationships were analyzed using a series of kinetic equations. In order to achieve the possibility of comparing the quality of fit of various equations, all optimizations were carried out by minimizing the sum of the squares of concentration deviations from the kinetic dependence expressed as a c_teor_(t) profile

(2) $$\min\left(\sum_{i=1}^{n}{\left[c_{i}-c_{teor}(t_{i})\right]}^{2}\right)$$

where the following are defined: c—concentration; t—time; and i—point number.

In the first stage of the analysis of the kinetic data, they were summarized in the linear coordinates of the Bangham relationship (i.e., log(log(co/c)) ~ log t), which is used to test compliance with the model of intraparticle diffusion models (IDMs). It should be noted that for IDM, the slope is 0.5 [31].

The simplest equation represents the first-order kinetics (FOE/PFOE), which can be expressed as

(3) $$ln\left(a_{eq}-a\right)=lna_{eq}-k_{1}t$$

(the so-called Lagargren equation) or

(4) $$c=c_{eq}+(c_{o}-c_{eq})\exp(-k_{1}t)$$

where a denotes adsorption amount, indices “o” and “eq” denote the initial and equilibrium values, and k_1_ denotes coefficient adsorption rates [40,41]. The above equation gave a good fit for the adsorption kinetics of nitro derivatives of benzoic acid. However, the best in this respect turned out to be the multi-exponential equation (m-exp), which describes parallel first-order processes:

(5) $$c=(c_{o}-c_{eq})\sum_{i=1}^{n}f_{i}\exp(-k_{i}t)+c_{eq}$$

or

(6) $$c=c_{o}-c_{o}u_{eq}\sum_{i=1}^{n}f_{i}[1-\exp(-k_{i}t)]$$

and

(7) $$\sum_{i=1}^{n}f_{i}=1$$

where the following are defined: f_i_—normalized share in the kinetics of process “i”; k_i_—rate coefficient; and u_eq_ = 1 − c_eq_/c_o_—relative loss of adsorbate from the solution [42].

To analyze the experimental data, the second-order kinetics equations (SOE/PSOE) [41,42,43] were also used, which can be expressed as:

(8) $$a=a_{eq}\frac{k_{2}t}{1+k_{2}t}$$

or as linear forms

(9) $$\frac{t}{a}=\frac{1}{a_{eq}}\left(\frac{1}{k_{2}t}+t\right)$$

and

(10) $$a=a_{eq}-\frac{1}{k_{2}}\left(\frac{a}{t}\right)$$

where k_2a_ is the rate coefficient of pseudo-second order kinetics and k_2_ = k_2a_a_eq_. This equation gave a good fit for the adsorption kinetics of benzoic acid. 

For all measurement series, the applicability of the 1,2-mixed-order kinetic equation (MOE), which is a generalization of first- and second-order kinetics, was also analyzed. Mathematically, this equation is identical to the IKL equation, which corresponds to Langmuir kinetics. However, it should be mentioned that MOE is an empirical equation and its parameters do not have to be related to the parameters of the Langmuir isotherm. MOE kinetics can be represented as the relative progress of F adsorption over time:

(11) $$F=a/a_{eq}=\frac{1-\exp(-k_{1}t)}{1-f_{2}\exp(-k_{1}t)}$$

or

(12) $$\ln\left(\frac{1-F}{1-f_{2}F}\right)=-k_{1}t$$

where the following are defined: f_2_ < 1—normalized contribution to the kinetics of the second-order process; k_1_—kinetic coefficient for the partial first-order process. The above equation gave very good fits for all experimental systems [44,45].

Finally, the data were analyzed using a kinetic equation equivalent to the MOE equation and taking into account non-ideal effects—the fractal-like MOE equation (f-MOE). It has the form:

(13) $$F=\frac{{1-exp\left(-k_{1}t\right)}^{p}}{{1-f_{2}exp\left(-k_{1}t\right)}^{p}}$$

where p—the fractal coefficient. In some cases, f-MOE can simplify to the equations: MOE (p = 0), f-FOE (f_2_ = 0), and f-SOE (f_2_ = 1) [45,46].

Half-times t_1/2_ were also determined for each measurement series, which can be defined as the times needed to achieve the half-concentration c_1/2_ = (c_o_ + c_eq_)/2. For the FOE equation and for individual m-exp terms, this parameter is determined from the relationship: $t_{1/2}=\ln2/k_{1}$; for PSOE: t_1/2_ = 1/k_2_; for MOE: $t_{1/2}=\ln(2-f_{2})/k_{1}$; and for m-exp, this quantity should be determined numerically.

#### 4.2.3. Thermal Analysis

Thermal analysis was performed using a QMS 403D Aelos working in combination with a STA449F1 Jupiter mass spectrometer (Netzsch, Selb, Germany) and TGA-IR Tensor 27 (Bruker, Billerica, MA, USA). Prepared samples (20 mg) of pure GAC and carbon after adsorption of aromatic organic acids were placed in a corundum crucible and heated at a rate of 10 °C/min in the temperature range from 30 to 950 °C. Measurements were performed in a nitrogen atmosphere with a flow rate of 25 mL/min.

#### 4.2.4. XPS

X-ray photoelectron spectroscopy (XPS) measurements were performed for hybrid materials—GAC activated carbon after adsorption of nitrobenzoic acids. For this purpose, samples, on a molybdenum support, were introduced into the loading lock of the ultra-high vacuum system (Prevac). Later, they were degassed at room temperature to obtain a pressure of 7.5 × 10^−8^ mbar. The sample was then placed in the XPS analysis chamber. Measurements were performed under vacuum conditions of 7.5 × 10^−9^ mbar. A VG Scienta SAX 100 X-ray tube equipped with an aluminum anode emitting radiation with a characteristic line of Kα-Al 1486.6 eV was used to excite the samples. The VG Scienta XM 780 monochromator provided a radiation bandwidth of 0.2 eV. Data processing analysis and curve fitting (line shape: Gauss–Lorenz function combination) with Shirley-type background ablation were performed using CasaXPS software (version 2.3.23).

## 5. Conclusions

Equilibrium and kinetic studies of the adsorption of neutral and ionic forms of benzoic, 2-nitrobenzoic, 3-nitrobenzoic, and 4-nitrobenzoic acids and methylene blue from aqueous solutions on granular activated carbon (GAC) were carried out. 

A strong dependence of the amount and rate of adsorption on the pH of the solution was demonstrated. In the case of sorption of aromatic organic acids, the highest efficiency of the adsorption process was observed at pH = 2. In a strongly acidic environment, the BA, 2-NBA, 3-NBA, and 4-NBA molecules have a molecular form, which eliminates the effect of competitive adsorption for the active site of GAC activated carbon between hydroxyl ions and anions originating from the dissociation of selected adsorbates. The obtained maximum adsorption amounts were 11.59 mmol/g, 6.57 mmol/g, 9.38 mmol/g, and 2.70 mmol/g for BA, 2-NBA, 3-NBA, and 4-NBA, respectively.

In the case of methylene blue adsorption, the highest adsorption efficiency was observed at pH = 12, due to additional electrostatic attraction interactions between the negatively charged surface of activated carbon (GAC) and the dye cations. Under strongly alkaline conditions, an adsorbed amount of 0.24 mmol/g was obtained. 

The generalized Langmuir isotherm was used to analyze the adsorption equilibrium data. The coefficient of determination R^2^ ranged from 0.932 to 0.995, and the standard deviation SD(a) ranged from 0.012 to 0.096. For three of all the tested experimental systems, the full form of the GL isotherm equation was used. For the rest, simplified forms were used—the generalized Freundlich isotherm and the Tóth isotherm. In practice, this means that medium energy heterogeneity effects were observed for the adsorption of BA, 2-NBA, 3-NBA, 4-NBA, and MB on GAC.

The compilation of data in linear Bagnham relationships allowed for a preliminary assessment of the adsorption mechanism in the tested experimental systems. It has been shown that the IDM diffusion model cannot be used in this case. Therefore, the equations FOE, SOE, MOE, f-FOE, f-SOE, f-MOE, and m-exp were used to further analyze the kinetic data. The best quality fit of adsorption rate data was obtained using the multi-exponential equation (1 − R^2^ = (6.3 × 10^−6^)–(2.1 × 10^−4^)), with very good and good fits for the 1,2-order mixed equation (1 − R^2^ = (1.1 × 10^−5^)–(9.6 × 10^−3^)). 

Data obtained from thermal analysis measurements in a nitrogen atmosphere showed that the decomposition processes of hybrid materials occur in three stages. Moreover, it was observed that 3-NBA and 4-NBA were most strongly bound to the surface of activated carbon (GAC), for which the maximum desorption and defragmentation temperatures were 339.9 °C and 341.3 °C, respectively.

Finally, based on the data obtained using the XPS technique, it was found that in the case of the formation of a chemical bond with the surface groups of GAC activated carbon, the carboxyl group of nitrobenzoic acid is most often involved (practically 70%).

The presented research results determine the efficiency of adsorption of organic environmental pollutants in diverse pH environments. The obtained relationships can be a basis for optimizing the water and sewage treatment process using adsorption methods. In the future, comprehensive adsorption studies are planned in similar systems, but using new, cheaper, and at least as effective adsorbents.

## Figures and Tables

**Table 1:** Parameters of the generalized Langmuir eq. describing adsorption of BA, 2-, 3-, and 4-NBA and MB from dilute aqueous solutions on GAC activated carbon at different solution pH values.

System	Isotherm Type	a_m_^a^	m ^b^	n ^b^	logK ^c^	R^2 d^	SD(a) ^e^
BA/GAC pH = 2	GF	11.68	0.88	1	−0.07	0.950	0.039
BA/GAC pH = 4	GF	10.49	0.91	1	0.02	0.964	0.052
BA/GAC pH = 6	GF	6.86	0.88	1	−0.23	0.991	0.018
2-NBA/GAC pH = 2	GF	6.59	0.59	1	−0.70	0.985	0.023
2-NBA/GAC pH = 4	T	5.86	1	0.11	−0.23	0.995	0.012
2-NBA/GAC pH = 6	T	4.82	1	0.89	0.76	0.990	0.019
3-NBA/GAC pH = 2	GF	9.84	0.52	1	−0.77	0.990	0.019
3-NBA/GAC pH = 4	T	6.74	1	0.40	6.34	0.955	0.043
3-NBA/GAC pH = 6	T	5.47	1	0.85	−0.03	0.994	0.013
4-NBA/GAC pH = 2	GF	3.14	0.44	1	−2.56	0.969	0.049
4-NBA/GAC pH = 4	GL	2.69	0.62	0.91	0.43	0.969	0.049
4-NBA/GAC pH = 6	T	2.03	1	0.15	7.59	0.962	0.056
MB/GAC pH = 2	GL	0.17	0.47	0.27	0.14	0.979	0.033
MB/GAC pH = 7	GL	0.22	0.43	0.94	1.15	0.990	0.019
MB/GAC pH = 12	T	0.29	1	0.31	4.20	0.932	0.096

a_m_^a^—adsorption capacity; m and n ^b^—parameters of energetic heterogeneity; logK ^c^—logarithm of the adsorption equilibrium constant; R^2 d^—determination coefficient; and SD(a) ^e^—standard deviation.

**Table 2:** Relative standard deviations SD(c)/c_o_ (%) for m-exp, FOE, SOE, MOE, f-FOE, f-SOE, and f-MOE equations for BA, 2-, 3-, and 4-NBA, and MB adsorption on GAC activated carbon at different solution pH values.

System	m-exp [%]	FOE [%]	SOE [%]	MOE [%]	f-FOE [%]	f-SOE [%]	f-MOE [%]
BA/GAC pH = 2	0.100	2.348	0.331	0.089	0.861	0.217	0.218
BA/GAC pH = 4	0.076	2.877	0.272	0.273	0.969	0.129	0.130
BA/GAC pH = 6	0.163	2.106	0.965	0.301	0.519	0.736	0.739
2-NBA/GAC pH = 2	0.188	0.957	4.742	0.258	0.306	1.722	20.045
2-NBA/GAC pH = 4	0.174	2.183	1.574	0.381	0.977	0.477	18.709
2-NBA/GAC pH = 6	0.181	2.465	1.197	1.203	0.696	0.397	0.399
3-NBA/GAC pH = 2	0.437	0.860	4.097	0.435	0.545	1.225	17.176
3-NBA/GAC pH = 4	0.120	0.782	5.372	0.117	0.233	2.025	19.184
3-NBA/GAC pH = 6	0.159	3.940	0.788	0.792	0.876	0.633	0.164
4-NBA/GAC pH = 2	0.175	0.418	5.854	0.186	0.266	2.040	2.053
4-NBA/GAC pH = 4	0.083	0.765	5.602	0.106	0.150	2.225	19.236
4-NBA/GAC pH = 6	0.169	3.246	1.490	0.324	0.821	0.958	0.944
MB/GAC pH = 2	0.344	1.180	4.797	0.704	0.862	1.225	1.228
MB/GAC pH = 7	0.166	3.964	2.034	1.801	1.535	2.751	2.765
MB/GAC pH = 12	0.298	0.308	5.832	0.295	2.085	4.051	2.914

**Table 3:** The optimized parameters of multi-exponential eq. for BA, 2-, 3-, and 4-NBA and MB adsorption kinetics on GAC activated carbon at different solution pH values.

System	f_1_^a^, log k_1_^b^	f_2_^a^, log k_2_^b^	f_3_^a^, log k_3_^b^	u_eq_^c^	t_1/2_^d^ [min]	SD(c)/c_o_^e^ [%]	1 − R^2 f^
BA/GAC pH = 2	0.306; −1.90	0.476; −2.44	0.218; −3.05	0.712	96.7	0.100	1.9 × 10^−5^
BA/GAC pH = 4	0.240; −1.80	0.487; −2.39	0.273; −3.09	0.739	169.0	0.076	1.1 × 10^−5^
BA/GAC pH = 6	0.137; −1.33	0.579; −2.09	0.284; −2.7	0.739	257.8	0.163	5.5 × 10^−5^
2-NBA/GAC pH = 2	0.112; −1.82	0.846; −2.41	0.041; −3.43	1	144.0	0.188	3.2 × 10^−5^
2-NBA/GAC pH = 4	0.324; −1.95	0.509; −2.41	0.167; −2.98	0.962	160.6	0.174	3.2 × 10^−5^
2-NBA/GAC pH = 6	0.248; −1.76	0.403; −2.58	0.349; −3.56	0.456	310.0	0.181	2.1 × 10^−4^
3-NBA/GAC pH = 2	0.218; −1.83	0.782; −2.25	-	0.957	99.2	0.437	2.1 × 10^−3^
3-NBA/GAC pH = 4	0.046; −1.69	0.439; −2.16	0.515; −2.45	0.994	132.9	0.120	1.3 × 10^−5^
3-NBA/GAC pH = 6	0.129; −1.78	0.463; −2.48	0.408; −3.19	0.962	311.9	0.159	2.5 × 10^−5^
4-NBA/GAC pH = 2	0.319; −2.27	0.681; −2.56	-	0.996	133.5	0.175	2.9 × 10^−5^
4-NBA/GAC pH = 4	0.01; −1.03	0.238; −2.01	0.752; −2.39	0.997	199.9	0.083	6.3 × 10^−6^
4-NBA/GAC pH = 6	0.185; −1.91	0.508; −2.52	0.307; −3.11	0.982	249.3	0.169	2.7 × 10^−5^
MB/GAC pH = 2	0.731; −2.53	0.269; −3.04	-	1	395.5	0.344	1.02 × 10^−4^
MB/GAC pH = 7	0.099; 0.53	0.837; −2.88	0.064; −2.17	0.997	307.2	0.166	3.51 × 10^−5^
MB/GAC pH = 12	0.017; −2.1	0.983; −2.58	-	0.992	260.2	0.298	7.54 × 10^−5^

f_1_, f_2_, and f_3_^a^—the terms of m-exp equation; log k_1_, log k_2,_ and log k_3_^b^—logarithm of the rate constant; u_eq_^c^—the relative loss of adsorbate from the solution; t_1/2_^d^—half-time; SD(c)/c_o_^e^—relative standard deviation; and 1 − R^2 f^—indetermination coefficient.

**Table 4:** The optimized parameters of the 1,2-mixed-order eq. for BA, 2-, 3-, and 4-NBA and MB adsorption kinetics on GAC activated carbon at different solution pH values.

System	f_2_ ^a^	log k_1_ ^b^	u_eq_ ^c^	t_1/2_ ^d^ [min]	SD(c)/c_o_ ^e^ [%]	1 − R^2 f^
BA/GAC pH = 2	0.884	−3.16	0.713	97.0	0.089	1.6 × 10^−5^
BA/GAC pH = 4	0.998	−4.87	0.756	158.0	0.273	1.4 × 10^−4^
BA/GAC pH = 6	0.827	−2.78	0.738	181.3	0.301	1.9 × 10^−4^
2-NBA/GAC pH = 2	0.355	−2.49	0.981	140.0	0.258	3.2 × 10^−5^
2-NBA/GAC pH = 4	0.722	−2.76	0.952	155.6	0.381	1.6 × 10^−4^
2-NBA/GAC pH = 6	0.997	−2.44	0.407	274.6	1.203	9.6 × 10^−3^
3-NBA/GAC pH = 2	0.302	−2.26	0.958	99.4	0.435	2.1 × 10^−3^
3-NBA/GAC pH = 4	0.302	−2.40	0.992	132.6	0.117	1.4 × 10^−5^
3-NBA/GAC pH = 6	0.997	−5.0	0.997	342.7	0.792	6.3 × 10^−4^
4-NBA/GAC pH = 2	0.174	−2.52	0.992	134.0	0.186	3.3 × 10^−5^
4-NBA/GAC pH = 4	0.295	−2.4	0.998	198.8	0.106	1.1 × 10^−5^
4-NBA/GAC pH = 6	0.889	−3.38	0.992	254.2	0.324	1.0 × 10^−4^
MB/GAC pH = 2	0.323	−2.77	0.984	443.1	0.704	4.4 × 10^−4^
MB/GAC pH = 7	0.423	−2.99	1	304.9	1.801	7.9 × 10^−5^
MB/GAC pH = 12	0.038	−2.59	0.994	260.2	0.295	7.6 × 10^−5^

f_2_ ^a^—the normalized share of the second order process in the kinetics; log k_1_ ^b^—logarithm of the rate constant; u_eq_ ^c^—the relative loss of adsorbate from the solution; t_1/2_ ^d^—half-time; SD(c)/c_o_ ^e^—relative standard deviation; and 1 − R^2 f^—indetermination coefficient.

**Table 5:** The thermal decomposition data of pure GAC activated carbon and after BA, 2-, 3-, and 4-NBA adsorption.

Sample		TG		DTG	DSC
	ΔT [°C]	Mass Loss [%]	Total Mass Loss [%]	Tmin [°C]	endo/exo
GAC	30–180	0.48	3.03	52	endo
	180–1000	2.55		292.3 697.5	exo exo
BA/GAC	30–180	0.36	18.77	110.4	endo
	180–500	11.02		299.4	endo
	500–1000	7.37		733.6	endo
2-NBA/GAC	30–180	0.44	23.39	119.4	endo
	180–500	7.53		294.4 444.9	exo exo
	500–1000	15.37		711.3	exo
3-NBA/GAC	30–180	0.42	15.40	104.9	endo
	180–500	7.69		339.9 444.9	exo exo
	500–1000	7.25		719.9	exo
4-NBA/GAC	30–180	0.38	17.38	121.3	endo
	180–500	8.17		341.3 410.3	exo exo
	500–1000	8.81		741.3	exo

**Table 6:** M/Z values in the mass spectrum for pure GAC activated carbon and after BA, 2-, 3-, and 4-NBA adsorption.

	Sample		
	GAC	BA/GAC	2-, 3-, 4-NBA/GAC
***m*/*z***	17	17	17
	18	18	18
	44	39	30
	78	44	39
		45	44
		50	45
		51	46
		65	50
		75	51
		78	65
		105	75
		122	78

**Table 7:** XPS survey spectra results and the determined elemental compositions (as atomic%) for 2-NBA/GAC, 3-NBA/GAC, and 4-NBA/GAC.

Sample	Name	Peak Position [eV]	Full Width at Half Maximum (FWHM)	Atomic Concentration [at.%]
2-NBA/GAC	C 1s	284.8	2.5	92.5
	N 1s	407.1	2.5	0.8
	O 1s	533.1	3.4	6.6
3-NBA/GAC	C 1s	284.8	2.5	86.3
	N 1s	406.3	2.6	1.5
	O 1s	533.1	4.6	12.2
4-NBA/GAC	C 1s	284.8	2.5	91.3
	N 1s	405.6	2.1	1.2
	O 1s	532.3	3.8	7.5

**Table 8:** C 1s, O 1s and N 1s in various chemical combinations and their atomic concentration for 2-NBA/GAC, 3-NBA/GAC, and 4-NBA/GAC.

Sample	Name	Peak Position [eV]	Full Width at Half Maximum (FWHM)	Atomic Concentration [at.%]	Suggested Binding
2-NBA/GAC	C 1s A	284.5	0.80	90.6	C=C sp2
	C 1s B	285.00	1.24	0.0	C-C/C-H
	C 1s C	286.20	1.24	3.6	C-OH/C-O-C
	C 1s D	287.30	1.24	3.0	C=O
	C 1s E	288.70	1.24	2.8	O=C-O-
	O 1s A	530.32	1.45	5.1	quinones
	O 1s B	531.63	1.75	27.1	O=C/O=C-O-R
	O 1s C	533.05	1.64	51.9	Ar-OH
	O 1s D	534.76	1.67	15.9	O=C-O-R
	N 1s A	399.06	1.59	15.3	organic matrix
	N 1s B	401.12	1.59	18.0	organic matrix
	N 1s C	402.75	1.58	7.6	N-oxide
	N 1s D	405.93	1.48	59.1	nitro group
3-NBA/GAC	C 1s A	284.50	0.78	90.8	C=C sp2
	C 1s B	285.00	1.32	0.0	C-C/C-H
	C 1s C	286.20	1.32	4.0	C-OH/C-O-C
	C 1s D	287.30	1.32	2.2	C=O
	C 1s E	288.70	1.32	3.0	O=C-O-
	O 1s A	530.14	1.75	11.3	quinones
	O 1s B	531.69	1.75	27.6	O=C/O=C-O-R
	O 1s C	533.08	1.72	50.2	Ar-OH
	O 1s D	534.65	1.75	10.9	O=C-O-R
	N 1s A	399.04	1.71	17.0	organic matrix
	N 1s B	401.06	1.63	16.4	organic matrix
	N 1s C	402.67	1.59	5.3	N-oxide
	N 1s D	406.00	1.52	61.3	nitro group
4-NBA/GAC	C 1s A	284.50	0.78	91.1	C=C sp2
	C 1s B	285.00	1.29	0.0	C-C/C-H
	C 1s C	286.20	1.29	3.2	C-OH/C-O-C
	C 1s D	287.30	1.29	2.4	C=O
	C 1s E	288.80	1.29	3.2	O=C-O-
	O 1s A	530.24	1.47	7.8	quinones
	O 1s B	531.75	1.75	30.5	O=C/O=C-O-R
	O 1s C	533.03	1.75	47.4	Ar-OH
	O 1s D	534.64	1.72	14.3	O=C-O-R
	N 1s A	399.24	1.80	15.9	organic matrix
	N 1s B	401.06	1.72	19.4	organic matrix
	N 1s C	402.66	1.64	8.0	N-oxide
	N 1s D	405.92	1.60	56.7	nitro group

**Table 9:** Chosen physicochemical properties of the studied organic compounds [3,4,5,6,7].

Adsorbate	Structural Formula	M ^a^ [g/mol]	c_s_^b^ [g/L]	pK_a_ ^c^	m.p. ^d^ [°C]	b.p. ^e^ [°C]	Chemical Safety
Benzoic acid	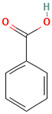	122.12	2.9	4.2	122.4	249.2	Corrosive Health Hazard
2-Nitrobenzoic acid	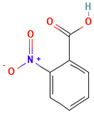	167.12	6.8	2.17	147.5	295.7	Irritant
3-Nitrobenzoic acid	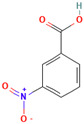	167.12	3.0	3.45	140–141	295.7	Irritant
4-Nitrobenzoic acid	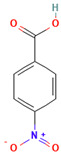	167.12	0.42	3.44	237–240	295.7	Irritant
Methylene blue	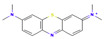	319.85	43.6	>12	100–110	-	Corrosive Irritant

M ^a^—molar mass; c_s_ ^b^—solubility in water at 25 °C; pK_a_ ^c^—ionization constant; m.p. ^d^—melting point; and b.p. ^e^—boiling point.

## Data Availability

The data and samples are available from the authors.

## Supplementary Materials

The following supporting information can be downloaded at: https://www.mdpi.com/article/10.3390/molecules29133032/s1, Figure S1: MS profiles of the main gaseous degradation products of GAC activated carbon measured in nitrogen atmosphere: OH (*m*/*z* = 17; (a)), H_2_O (*m*/*z* = 18; (b)), CO_2_ (*m*/*z* = 44; (c)), and C_6_H_6_ (*m*/*z* = 78; (d)); Figure S2: MS profiles of the main gaseous degradation products of GAC activated carbon after adsorption of benzoic acid measured in nitrogen atmosphere: OH (*m*/*z* = 17; (a)), H_2_O (*m*/*z* = 18; (b)), C_3_H_3_^+^ (*m*/*z* = 39; (c)), CO_2_ (*m*/*z* = 44; (d)), COOH^+^ (*m*/*z* = 45; (e)), C_4_H_2_^+^ (*m*/*z* = 50; (f)), C_4_H_3_^+^ (*m*/*z* = 51; (g)), C_5_H_5_^+^ (*m*/*z* = 65; (h)), C_6_H_3_^+^ (*m*/*z* = 75; (i)), C_6_H_6_ (*m*/*z* = 78; (j)), C_6_H_5_C=O^+^ (*m*/*z* = 105; (k)), and C_6_H_5_COOH (*m*/*z* = 122; (l)); Figure S3: MS profiles of the main gaseous degradation products of GAC activated carbon after adsorption of nitrobenzoic acids measured in nitrogen atmosphere: OH (*m*/*z* = 17; (a)), H_2_O (*m*/*z* = 18; (b)), NO (*m*/*z* = 30; (c)), C_3_H_3_^+^ (*m*/*z* = 39; (d)), CO_2_ (*m*/*z* = 44; (e)), COOH^+^ (*m*/*z* = 45; (f)), NO_2_ (*m*/*z* = 46; (g)), C_4_H_2_^+^ (*m*/*z* = 50; (h)), C_4_H_3_^+^ (*m*/*z* = 51; (i)), C_5_H_5_^+^ (*m*/*z* = 65; (j)), C_6_H_3_^+^ (*m*/*z* = 75; (k)), and C_6_H_6_ (*m*/*z* = 78; (l)).
