# Supplementary material for: Equilibrium and Kinetic Studies on Adsorption of Neutral and Ionic Species of Organic Adsorbates from Aqueous Solutions on Activated Carbon
Source: Molecules. 2024 Jun 26;29(13):3032. doi: 10.3390/molecules29133032 (PMC11243464; doi:10.3390/molecules29133032)
Supplement: Supplementary file 1 [file molecules-29-03032-s001.zip › molecules-3058473-supplementary.pdf]

Supplementary material for the paper:

Equilibrium and kinetic studies on adsorption of neutral and ionic species of organic adsorbates from aqueous solutions on activated carbon

Małgorzata Wasilewska*, Anna Derylo-Marczewska* and Adam W. Marczewski

Department of Physical Chemistry, Institute of Chemical Sciences, Faculty of Chemistry, Maria Curie-Sklodowska University in Lublin, Maria Curie-Sklodowska Sq. 3, 20-031 Lublin, Poland

***Corresponding authors:** e-mail: malgorzata.wasilewska@mail.umcs.pl, anna.derylo-marczewska@mail.umcs.pl

2.3. Thermal analysis

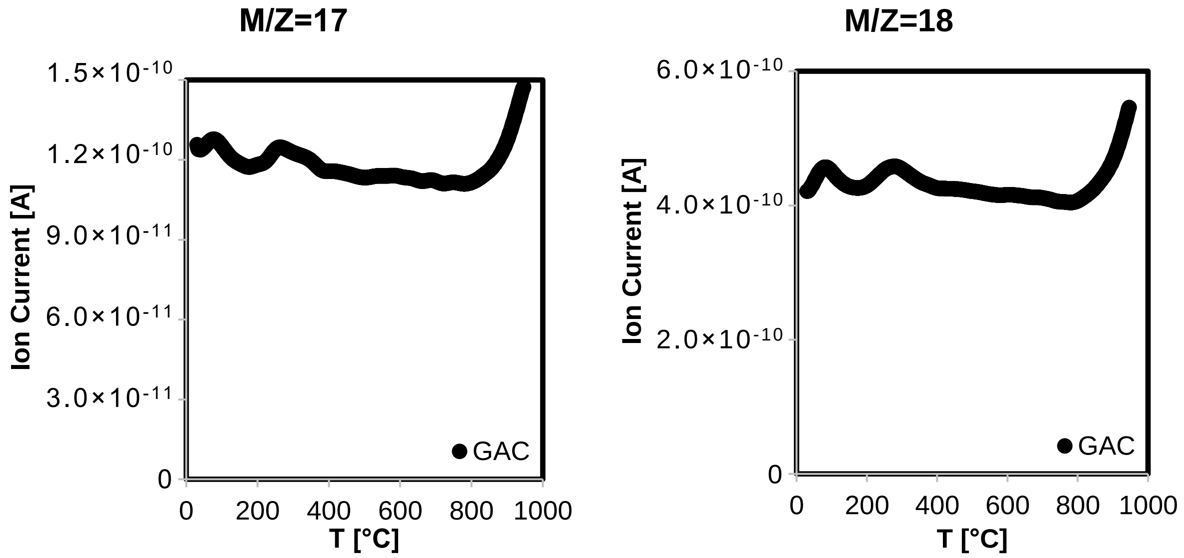

(**a**) (**b**)

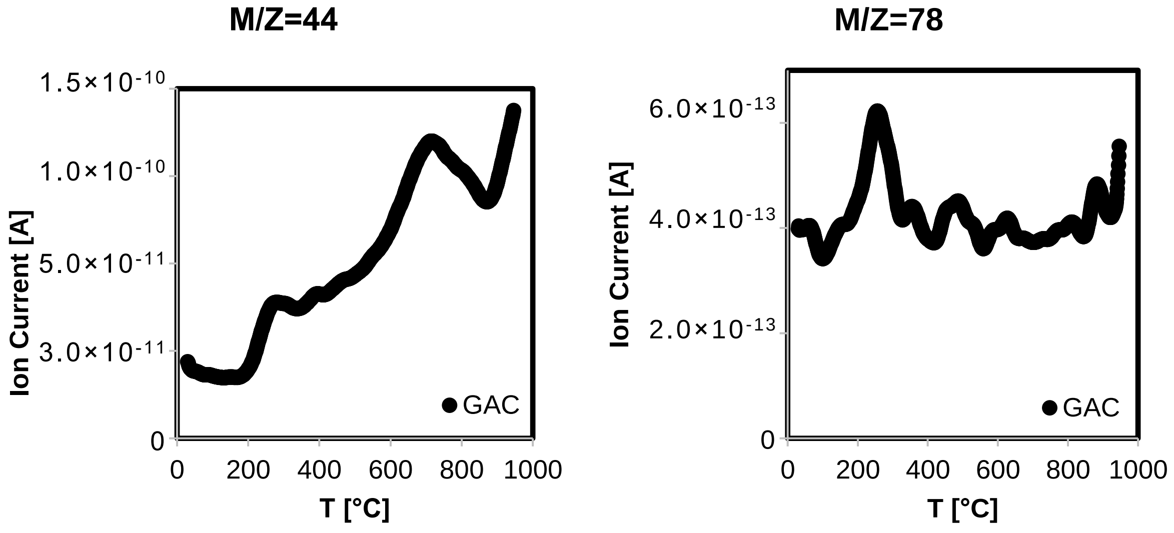

(**c**) (**d**)

Figure S1. MS profiles of the main gaseous degradation products of GAC activated carbon measured in nitrogen atmosphere: OH (m/z = 17; (**a**)) , H_2O (m/z = 18; (**b**)), CO_2 (m/z = 44; (**c**)) and C_6H_6 (m/z = 78; (**d**)).

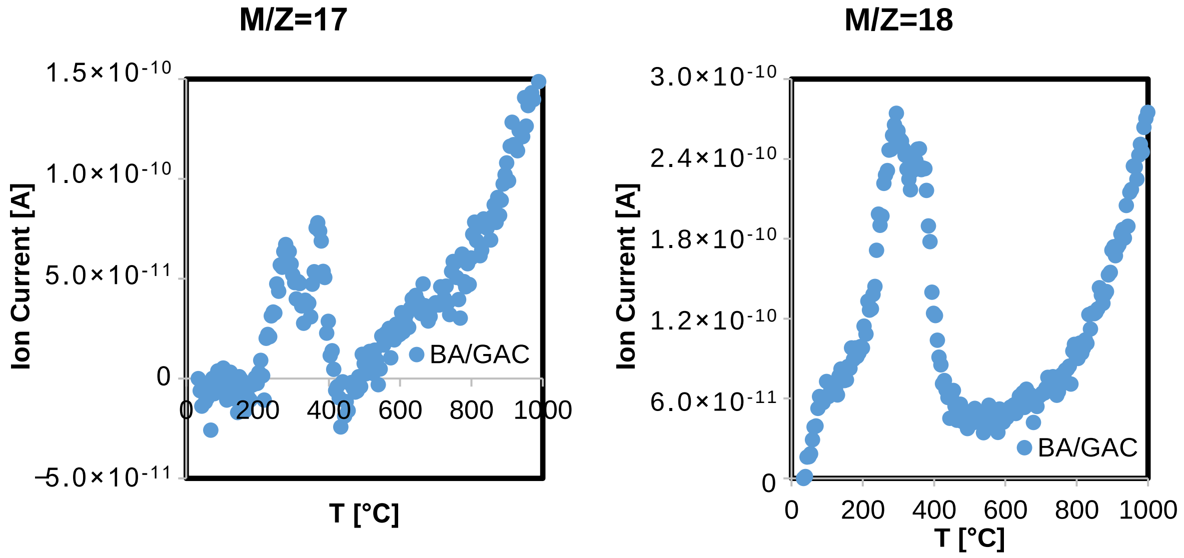

(**a**) (**b**)

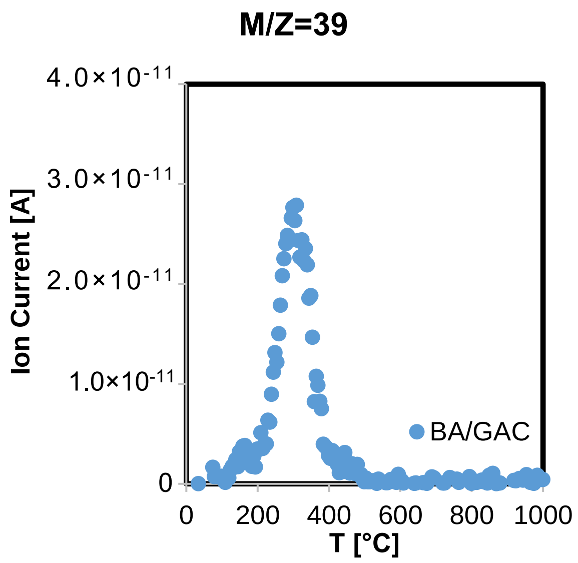
M/Z=39
Ion Current [A]
4.0×10-11
3.0×10-11
2.0×10-11
1.0×10-11
0
0
200
400
600
800
1000
T [°C]
BA/GAC

(c)

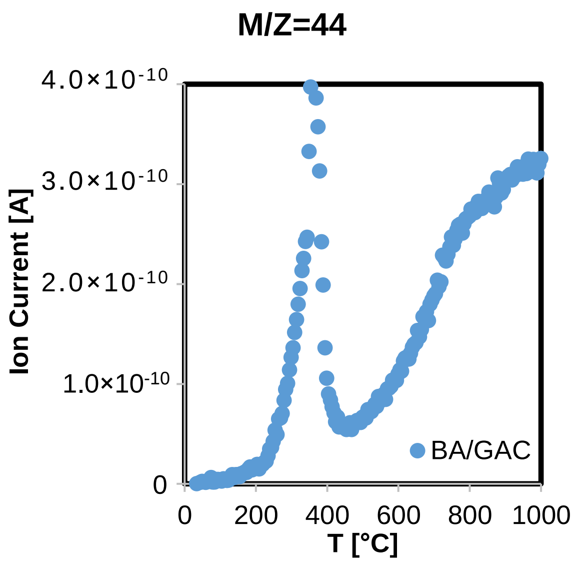
M/Z=44
Ion Current [A]
4.0×10-10
3.0×10-10
2.0×10-10
1.0×10-10
0
0
200
400
600
800
1000
T [°C]
BA/GAC

(d)

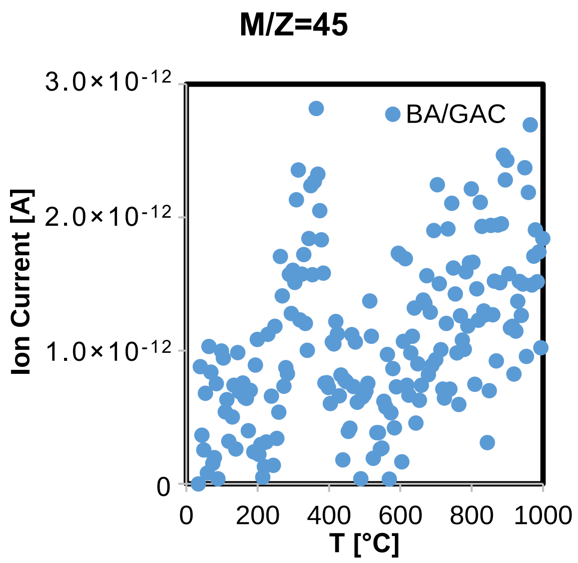
M/Z=45
Ion Current [A]
3.0×10-12
2.0×10-12
1.0×10-12
0
0
200
400
600
800
1000
T [°C]
BA/GAC

(e)

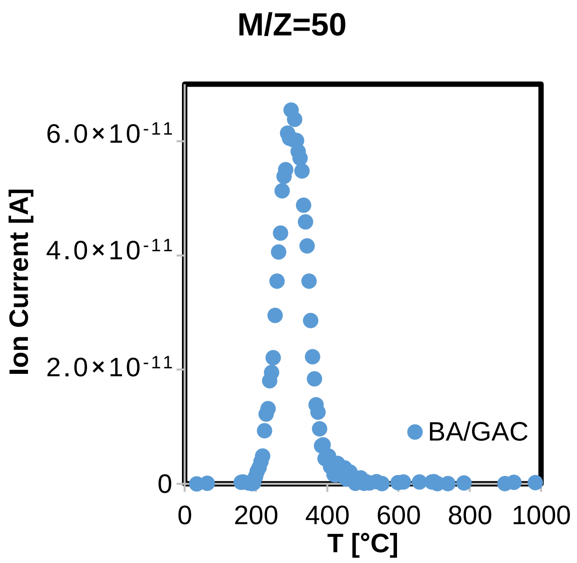
M/Z=50
Ion Current [A]
6.0×10-11
4.0×10-11
2.0×10-11
0
0
200
400
600
800
1000
T [°C]
BA/GAC

(f)

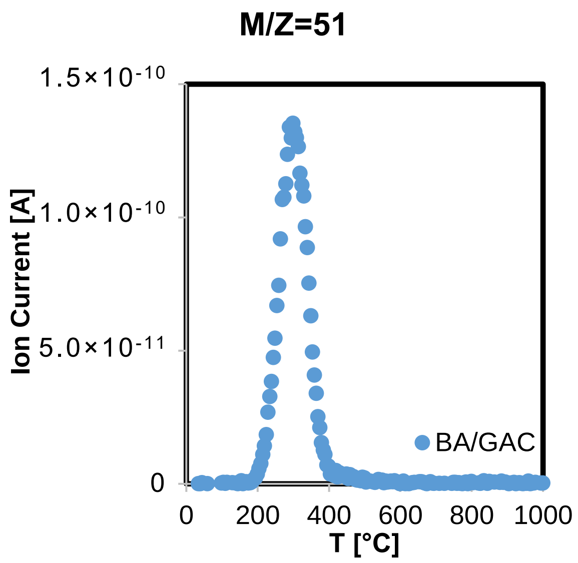
M/Z=51
1.5×10-10
1.0×10-10
5.0×10-11
0
Ion Current [A]
BA/GAC
0 200 400 600 800 1000
T [°C]

(g)

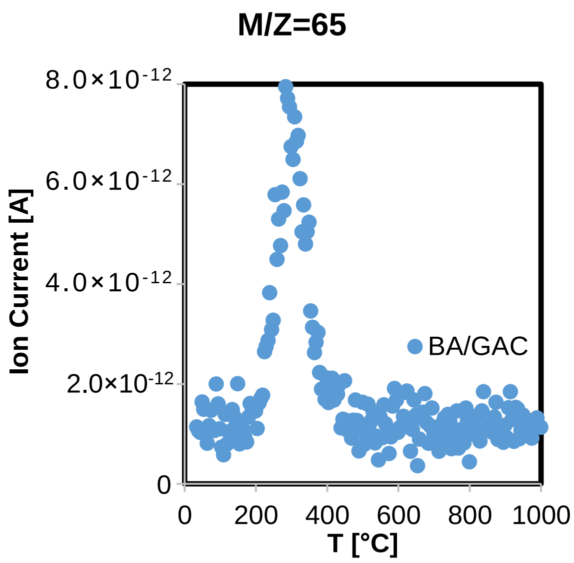
M/Z=65
8.0×10-12
6.0×10-12
4.0×10-12
2.0×10-12
0
Ion Current [A]
BA/GAC
0 200 400 600 800 1000
T [°C]

(h)

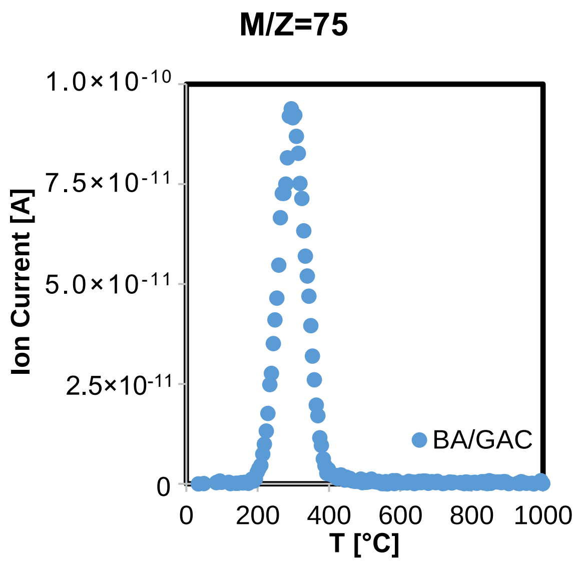
M/Z=75
1.0×10-10
7.5×10-11
5.0×10-11
2.5×10-11
0
Ion Current [A]
BA/GAC
0 200 400 600 800 1000
T [°C]

(i)

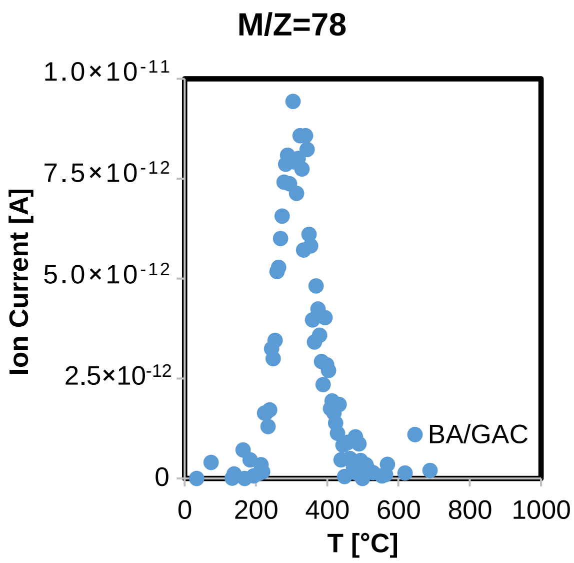
M/Z=78
1.0×10-11
7.5×10-12
5.0×10-12
2.5×10-12
0
Ion Current [A]
BA/GAC
0 200 400 600 800 1000
T [°C]

(j)

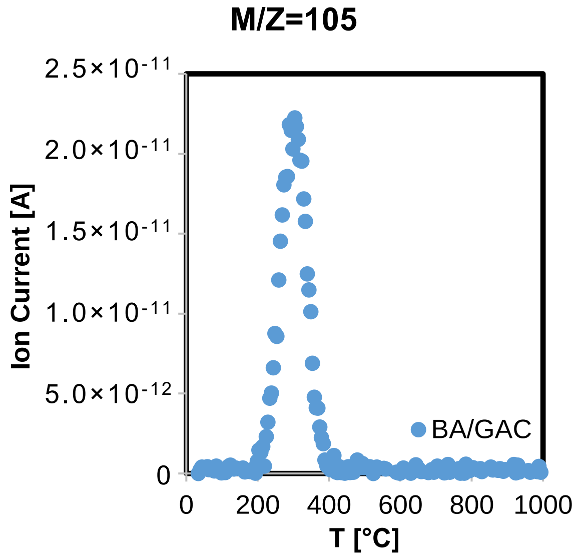

(**k**)

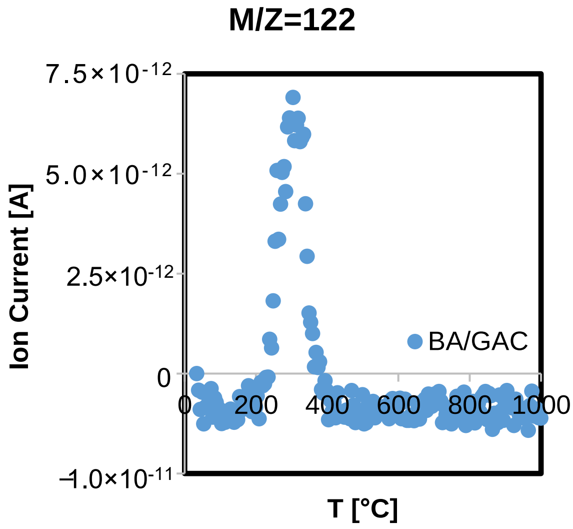

(**l**)

Figure S2. MS profiles of the main gaseous degradation products of GAC activated carbon after adsorption of benzoic acid measured in nitrogen atmosphere: OH (m/z = 17; (**a**)), H_2O (m/z = 18; (**b**)), $C_3H_3^+$ (m/z = 39; (**c**)), CO_2 (m/z = 44; (**d**)), $COOH^+$ (m/z = 45; (**e**)), $C_4H_2^+$ (m/z = 50; (**f**)), $C_4H_3^+$ (m/z = 51; (**g**)), $C_5H_5^+$ (m/z = 65; (**h**)), $C_6H_3^+$ (m/z = 75; (**i**)), C_6H_6 (m/z = 78; (**j**)), $C_6H_5C{=}O^+$ (m/z = 105; (**k**)) and C_6H_5COOH (m/z = 122; (**l**)).

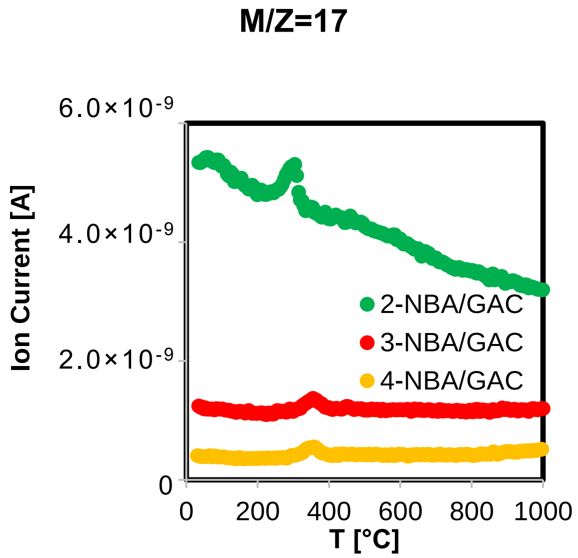

(**a**)

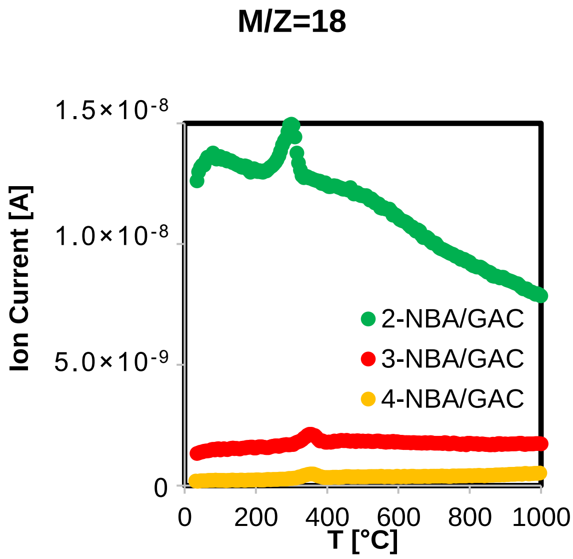

(**b**)

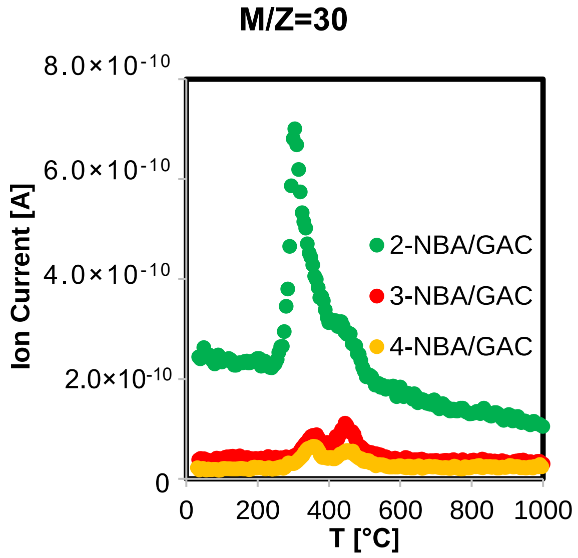
M/Z=30
Ion Current [A]
8.0×10-10
6.0×10-10
4.0×10-10
2.0×10-10
0
0 200 400 600 800 1000
T [°C]
2-NBA/GAC
3-NBA/GAC
4-NBA/GAC

(c)

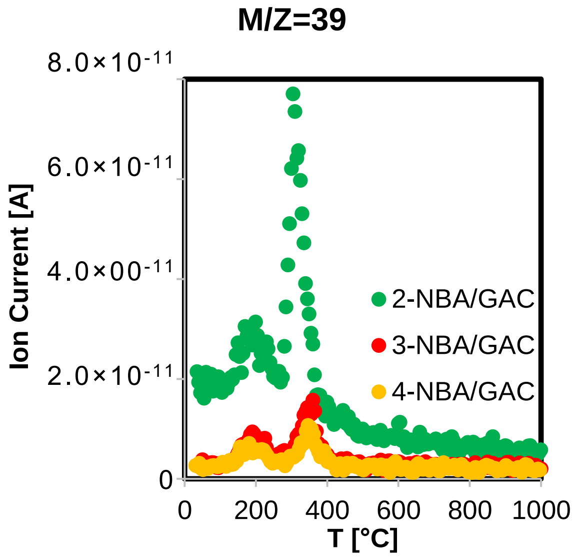
M/Z=39
Ion Current [A]
8.0×10-11
6.0×10-11
4.0×00-11
2.0×10-11
0
0 200 400 600 800 1000
T [°C]
2-NBA/GAC
3-NBA/GAC
4-NBA/GAC

(d)

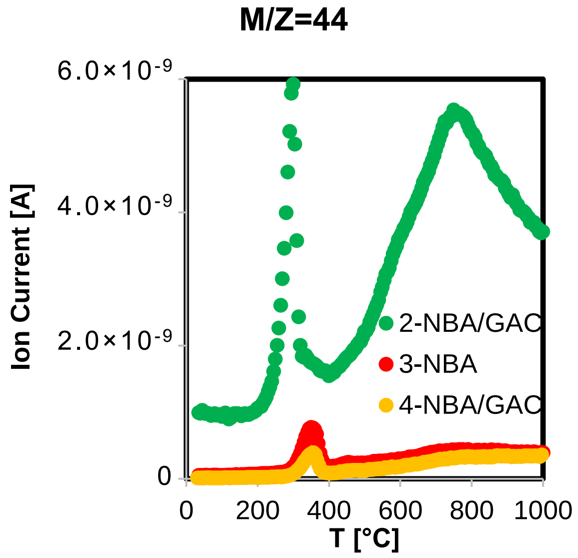
M/Z=44
Ion Current [A]
6.0×10-9
4.0×10-9
2.0×10-9
0
0 200 400 600 800 1000
T [°C]
2-NBA/GAC
3-NBA
4-NBA/GAC

(e)

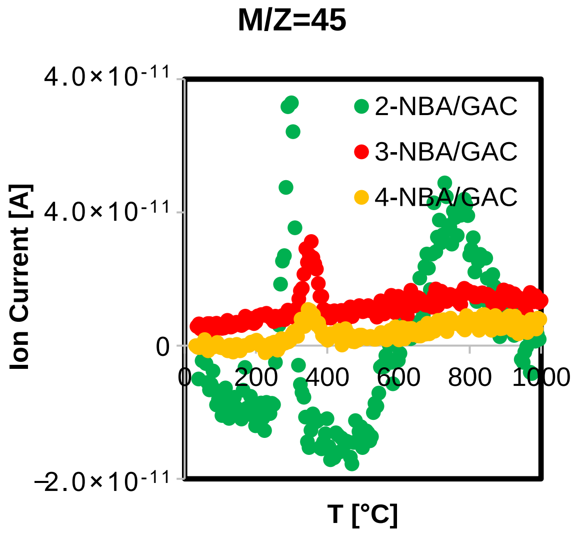
M/Z=45
Ion Current [A]
4.0×10-11
4.0×10-11
0
-2.0×10-11
0 200 400 600 800 1000
T [°C]
2-NBA/GAC
3-NBA/GAC
4-NBA/GAC

(f)

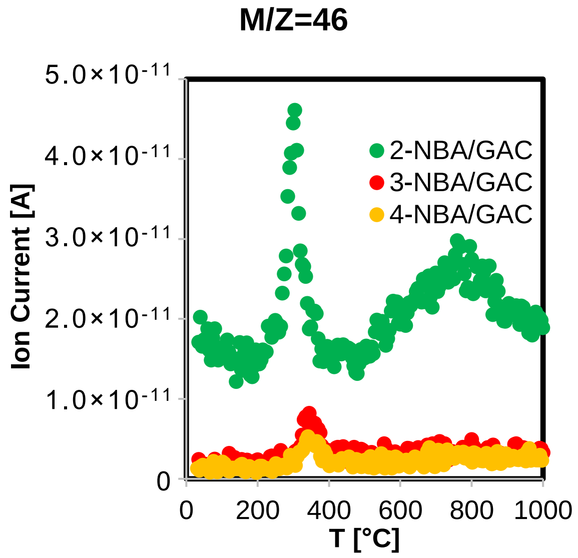
M/Z=46
5.0×10-11
4.0×10-11
3.0×10-11
2.0×10-11
1.0×10-11
0
Ion Current [A]
0
200
400
600
800
1000
T [°C]
2-NBA/GAC
3-NBA/GAC
4-NBA/GAC

(g)

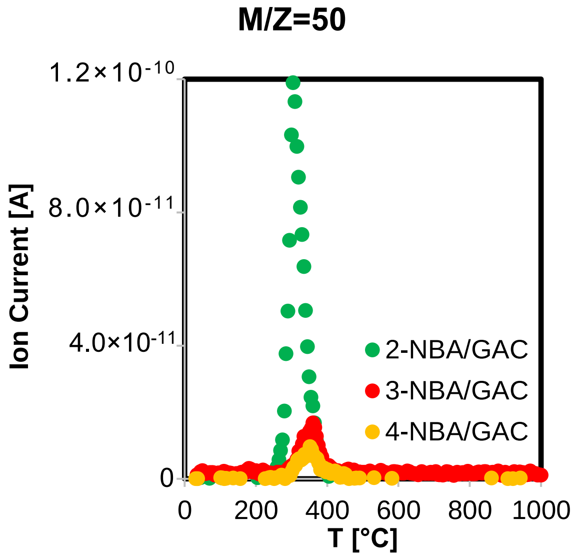
M/Z=50
1.2×10-10
8.0×10-11
4.0×10-11
0
Ion Current [A]
0
200
400
600
800
1000
T [°C]
2-NBA/GAC
3-NBA/GAC
4-NBA/GAC

(h)

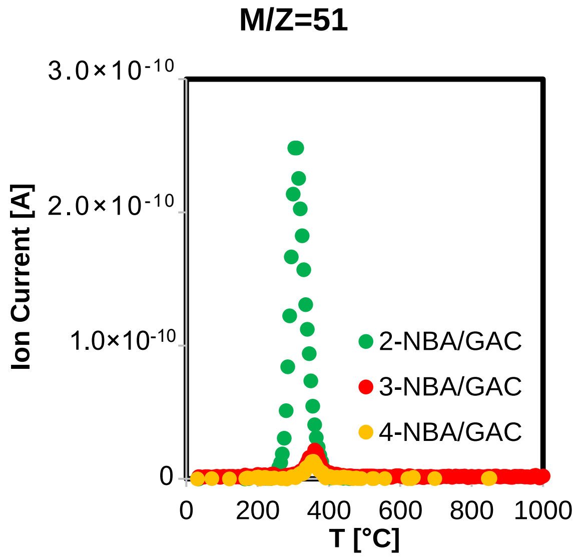
M/Z=51
3.0×10-10
2.0×10-10
1.0×10-10
0
Ion Current [A]
0
200
400
600
800
1000
T [°C]
2-NBA/GAC
3-NBA/GAC
4-NBA/GAC

(i)

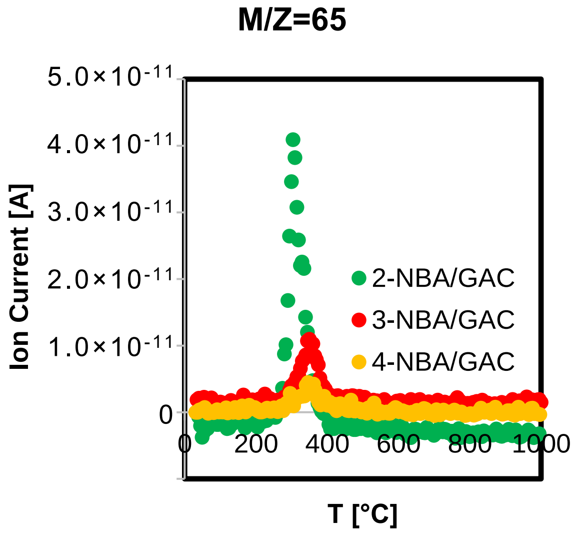
M/Z=65
5.0×10-11
4.0×10-11
3.0×10-11
2.0×10-11
1.0×10-11
0
Ion Current [A]
0
200
400
600
800
1000
T [°C]
2-NBA/GAC
3-NBA/GAC
4-NBA/GAC

(j)

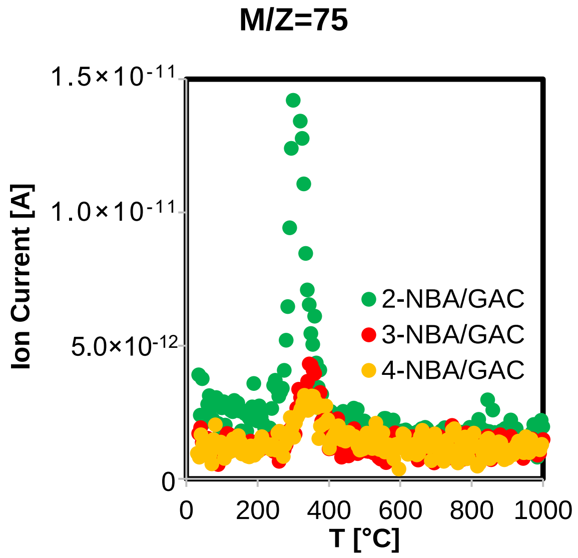

(**k**)

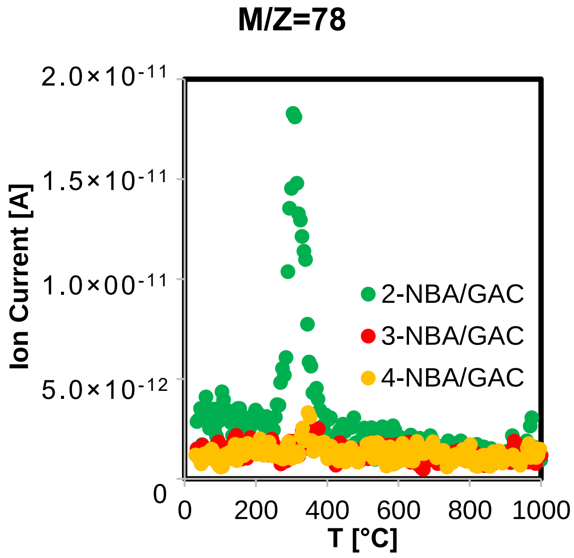

(**l**)

Figure S3. MS profiles of the main gaseous degradation products of GAC activated carbon after adsorption of nitrobenzoic acids measured in nitrogen atmosphere: OH (m/z = 17; (**a**)), H_2O (m/z = 18; (**b**)), NO (m/z = 30; (**c**)), $C_3H_3^+$ (m/z = 39; (**d**)), CO_2 (m/z = 44; (**e**)), $COOH^+$ (m/z = 45; (**f**)), NO_2 (m/z = 46; (**g**)), $C_4H_2^+$ (m/z = 50; (**h**)), $C_4H_3^+$ (m/z = 51; (**i**)), $C_5H_5^+$ (m/z = 65; (**j**)), $C_6H_3^+$ (m/z = 75; (**k**)) and C_6H_6 (m/z = 78; (**l**)).
